# A Fourteen Gene GBM Prognostic Signature Identifies Association of Immune Response Pathway and Mesenchymal Subtype with High Risk Group

**DOI:** 10.1371/journal.pone.0062042

**Published:** 2013-04-30

**Authors:** Arivazhagan Arimappamagan, Kumaravel Somasundaram, Kandavel Thennarasu, Sreekanthreddy Peddagangannagari, Harish Srinivasan, Bangalore C. Shailaja, Cini Samuel, Irene Rosita Pia Patric, Sudhanshu Shukla, Balaram Thota, Krishnarao Venkatesh Prasanna, Paritosh Pandey, Anandh Balasubramaniam, Vani Santosh, Bangalore Ashwathnarayanara Chandramouli, Alangar Sathyaranjandas Hegde, Paturu Kondaiah, Manchanahalli R. Sathyanarayana Rao

**Affiliations:** 1 Department of Neurosurgery, National Institute of Mental Health and Neuro Sciences, Bangalore, India; 2 Department of Microbiology and Cell Biology, Indian Institute of Science, Bangalore, India; 3 Department of Biostatistics, National Institute of Mental Health and Neuro Sciences, Bangalore, India; 4 Department of Neuropathology, National Institute of Mental Health and Neuro Sciences, Bangalore, India; 5 Sri SatyaSai Institute of Higher Medical Sciences, Bangalore, India; 6 Department of Molecular Reproduction, Development and Genetics, Indian Institute of Science, Bangalore, India; 7 Jawaharlal Nehru Centre for Advanced Scientific Research, Bangalore, India; Deutsches Krebsforschungszentrum, Germany

## Abstract

**Background:**

Recent research on glioblastoma (GBM) has focused on deducing gene signatures predicting prognosis. The present study evaluated the mRNA expression of selected genes and correlated with outcome to arrive at a prognostic gene signature.

**Methods:**

Patients with GBM (n = 123) were prospectively recruited, treated with a uniform protocol and followed up. Expression of 175 genes in GBM tissue was determined using qRT-PCR. A supervised principal component analysis followed by derivation of gene signature was performed. Independent validation of the signature was done using TCGA data. Gene Ontology and KEGG pathway analysis was carried out among patients from TCGA cohort.

**Results:**

A 14 gene signature was identified that predicted outcome in GBM. A weighted gene (WG) score was found to be an independent predictor of survival in multivariate analysis in the present cohort (HR = 2^.^507; B = 0^.^919; p<0^.^001) and in TCGA cohort. Risk stratification by standardized WG score classified patients into low and high risk predicting survival both in our cohort (p = <0^.^001) and TCGA cohort (p = 0^.^001). Pathway analysis using the most differentially regulated genes (n = 76) between the low and high risk groups revealed association of activated inflammatory/immune response pathways and mesenchymal subtype in the high risk group.

**Conclusion:**

We have identified a 14 gene expression signature that can predict survival in GBM patients. A network analysis revealed activation of inflammatory response pathway specifically in high risk group. These findings may have implications in understanding of gliomagenesis, development of targeted therapies and selection of high risk cancer patients for alternate adjuvant therapies.

## Introduction

Glioblastoma (GBM) is the most common and biologically aggressive brain tumor in adults. Despite standard therapeutic protocols, which include maximal surgical resection followed by radiation and chemotherapy with temozolomide, the prognosis of patients with GBM remains dismal, with median survival rates ranging from 12–17 months [1]. Some clinical variables such as patient age, preoperative Karnofsky performance score (KPS), and extent of resection, have been shown to be predictive of survival [1]–[3].These tumors demonstrate a marked heterogeneity in clinical behavior and recently, a lot of research is directed towards understanding the molecular and genetic basis for the pathogenesis and behaviorof GBM. There is also a need to identify robust prognostic indicators for efficient management of GBM. In this regard, genetic, epigenetic alterations, and expression of some genes have been correlated with poor or better prognosis in some of the recent studies [4], [5]. Among molecular biomarkers, the status of MGMT promoter methylation has been one of the most studied prognostic biomarkers of GBM [6].

Recent research is directed towards identification of gene signatures, comprising of multiple genes with varied functions, which can more accurately predict the behaviour of these tumors, facilitated by the availability of high throughput technologies to study a larger number of genes. Some studies have reported gene signatures which can be useful to classify various grades of glioma, classify subgroups in GBM or to identify prognostic subgroups in glioma[7]–[9]. Microarray based gene expression profiling of GBMs and gene specific studies with clinical correlation have identified few genes as molecular predictors of survival outcome [10]–[13]. Colman *et al.*, reported a 9 gene signature, derived by analyzing the data from four previously published data sets, which predicted patient survival outcome. They also suggested association of the signature with markers of glioma stem like cells, namely nestin and CD133 [10].However, due to the heterogeneity of these tumors, more robust prognostic gene signature panels are essential to improve the management of GBM. In view of this necessity, we have undertaken the present study, utilizing a cohort of patients of newly diagnosed GBM who were followed up prospectively. We have identified a 14 gene expression signature panel with a power to predict patient survival. Furthermore, this gene signature panel has been validated in an independent cohort of patients whose data is available through TCGA consortium data base.

## Methods

### Patient Population

This prospective study included a total number of 154 patients with histologically proven GBM who underwent surgical treatment at National Institute of Mental Health and Neurosciences and Sri SatyaSai Institute of Higher Medical Sciences, Bangalore, India between July 2006 and September 2009. This study has been approved by the ethics committee of NIMHANS (NIMHANS/IEC/No. RPA/060/05 dated 29.10.2005) and SSSIHMS (IEC No RPA/001/2005 dated 20.10.05) and patient’s written consent was obtained. All the patients were adults (age>18 yrs of age) with newly diagnosed GBM. Patients with previous surgery/recurrence were excluded from the study. All patients underwent total/near total excision of the tumor. Patients with post operative Karnofsky’s Performance Score (KPS) ≥70 were included in the study. Histological specimens were centrally reviewed and confirmed as GBM by the neuropathologist. All patients were treated subsequently with standard adjuvant therapy which included radiotherapy (total dose of 59.4 Gy, given in 33 fractions) with concomitant temozolomide (100 mg/day for 45 days), followed by five cycles of temozolomide at a dose of 150 mg/sq. m body surface area. Patients were followed up at regular intervals and their clinical status was documented. Overall survival was defined as the duration between surgery and death of the patient due to the disease. Of these 154 patients, gene expression data were available for 123 patients which were considered for further analysis.

### Tumor Samples

Tumor tissues were freshly collected from the neurosurgical operation theaters, bisected, and one half was fixed in 10% buffered neutral formalin, processed for paraffin sections, and was used for histopathology. The other half was placed in RNAlater (Ambion, Inc.) stored at −70°C and used for RNA isolation.

### RNA Isolation and RT-qPCR

Total RNA was extracted from frozen tissues by using TRI Reagent (Sigma, USA). The RNA samples were quantified by measuring the absorbance using a spectrophotometer and visualized on a MOPS-Formaldehyde gel for quality assurance. The relative quantitation of expression levels of selected genes was carried out using a two step strategy: in the first step, cDNA was generated from RNA derived from different tissue samples using cDNA Archive kit (ABI PRISM); subsequently real-time quantitative PCR was carried out in ABI PRISM 7900 (Applied Biosystems) sequence detection system with the cDNA as the template using gene specific primer sets and Dynamo kit containing SYBR green dye (Finnzyme, Finland). All measurements were made in triplicate. The genes GARS (glycyl-tRNAsynthetase), AGPAT1 (1-acylglycerol-3-phosphate O-acyltransferase 1), ATP5G1 (ATP synthase, H+ transporting, mitochondrial F0 complex, subunit C1 (subunit 9)) and RPL35A (ribosomal protein L35a) were used as internal controls as their expression levels were found to be unaltered in our previous array experiments. Normal brain tissue samples from epilepsy patients were used as control. Delta delta CT method was used for the calculation of ratios of gene expression. Sequences of RT-PCR primers used are given in **supplementary table S1**.

A total number of 175 genes (**Supplementary Table S2**) were selected for expression analysis and subsequent survival correlation. Amongst these 175 genes, 112 genes were selected based on their differential expression pattern in glioma as revealed by our previous microarray data [14]–[16]. These include genes that are differentially expressed in astrocytoma samples as compared to control normal brain tissues as well as for varying expression among GBMs. The remaining genes were selected from the literature as differentially regulated with prognostic value [17]. During this study, we validated the expression of selected 175 genes by real-time qPCR in an independent cohort of 123 GBM samples, which were prospectively selected and underwent uniform treatment as a part of this study. Since the values derived from real-time qPCR method are more accurate and reliable as compared to microarray derived expression values, we have used expression data for 175 genes from 123 GBM samples for the survival prediction.

### Immunohistochemistry

Analysis of protein expression was carried out by IHC for EGFR, CHI3L1/YKL-40, SOD2 and CALCRL (n = 123 for each marker). Antigen retrieval was done by heat treatment of the deparaffinized sections in a microwave oven for 30 minutes at 600 W in citrate buffer (10 mmol/L; pH 6.0). After the initial processing steps, sections were incubated overnight with primary antibody at 4°C (anti- CH13L1/YKL-40; Rabbit polyclonal antibody; 1∶500 dilution), anti-EGFR (Biogenex; E-30; 1∶50 dilution), anti- SOD2 (Sigma Life Science; HPA001814; 1∶300 dilution) and anti- CALCRL (Novus Biologicals;NBP1-85643; 1∶100 dilution). This was followed by incubation with secondary antibody (Biogenex; QD440-XAK for EGFR and CH13L1 and Thermo scientific [Ultravision Protein block] for SOD2 and CALCRL). The reaction was visualized by using 3, 3′-Diaminobenzidine (Sigma-Aldrich) as a chromogenic substrate. GBM tumors that showed elevated mRNA levels of EGFR, YKL-40, SOD2 and CALCRL respectively by qRT-PCR experiments served as positive controls. A negative control slide in which the primary antibody was excluded was incorporated with each batch of staining. A visual semiquantitative grading scale was applied to assess the intensity of the immunoreactivity as follows: zero (0) if the staining was absent, 1+ if it was weak, and 2+ if it was strong. Only2+ staining intensity was considered for analysis. The immunopositivity of EGFR, CHI3L1, SOD2 and CALCRL was assessed in more than 1,000 cells from each tumor specimen. The labeling index (LI) was expressed as a percentage of cells that showed 2+ positive staining among the total number of cells that were counted.

### Statistical Analysis and External Validation

One hundred and fifty four patients with GBM were prospectively recruited during the study period; gene expression data were available for 123 patients which were taken for further analysis. This group did not reveal any significant difference as compared to the whole cohort with respect to age, symptom duration, maximum follow up and median survival as studied by case bias analysis (**Supplementary Table S3**).

The mean age of the patient cohort was 46.5 years. The mean follow up period was 16.3±10.6 months (Median = 13 months; range = 1–47 months). At the time of analysis, 82 patients had expired due to the disease and 41 were alive. The median survival of this patient cohort was 16 months (95%CI: 13.37–18.62 months; Kaplan Meier analysis).

The expression patterns of 175 genes were obtained by qRT-PCR. The mRNA expression data of all the genes was used for the analysis. For identification of gene signature, the entire dataset of patients with complete information was considered. The expression levels of each gene in all the patients were expressed as log2 ratio for analysis. The missing values were imputed using a nearest neighbor algorithm [18], which uses k-nearest neighbors in the space of genes to impute missing expression values. We used 10 nearest neighbours imputation with minimum of 50% data for a given gene and 20% for a given subject. We used the supervised principal component method [19], available in the *superpc* package of R [20] with a Cox regression model [21] for survival to compute Wald scores for each gene. The genes were ranked based on their Wald scores. Utilizing the complete data set, we determined the optimal gene threshold by 40 fold cross-validation to select genes to optimize the score in supervised principal components of the selected gene expressions. The first supervised principal component of genes that reached a certain threshold of the score was computed and applied as a single explanatory variable in a Cox survival model. The adequacy of selected supervised principal component was tested by Likelihood ratio test for its significance. The data/feature reduction was achieved by Computing Cox regression scores (univariate) for all the 175 genes to form a reduced data matrix comprising only those features whose univariate coefficient exceeds a threshold value estimated by cross-validation. During the cross validation of the data, the program uses cross-validation method given by “pre-validation” approach of Tibshirani and Efron in supervised principal components [22]. The threshold was fixed at 0.85 which was obtained from the likelihood ratio value plot. With the selected threshold, a total of 14 genes were selected during the cross validation. The median rank and proportion of times appeared in the cross validation is shown (**Supplementary table S4**).

In order to compute prognostic score, the weightage corresponding to each gene was calculated by fitting multivariate Cox proportional hazards model. A weighted prognostic gene score (WG sore) was calculated using the following formula:

**WG score = ∑ (Cox regression coefficient X log2 ratio of each gene).**

The efficiency of the linear weighted score was also evaluated by calculating Area Under Receiver Operating Characteristic (ROC) curve with the time dependent censored survival data using Kaplan-Meier and spanning method [23]. Internal validation of WG score was done by randomly selecting 50% of the cases out from the total cohort. Further, a bootstrap internal validation was carried out to test the validity of weighted prognostic score. The power of the test was calculated by simulating 1000 bootstrap simulations. Statistical analysis was performed using SPSS version 15.0 and R software version 2.10.0 [20].

### External Validation with TCGA Data

The gene expression data (Agilent microarray platform) and clinical data from TCGA study [24] were accessed with prior permission and utilized to validate the impact of WG score on survival. The patients with proven GBM, with KPS>70 and lived at least for 30 days and received radiotherapy and some form of chemotherapy were chosen to form an external cohort for validation of the WG score derived from the present study. A total number of 130 patients who satisfied these criteria were considered for analysis. The mean age of the patients was 53.4 yrs (range: 17–82 yrs). The duration of follow up ranged from 1.3 to 110.3 months (mean = 21.9±21.97 months; median = 14.5 months). The gene expression levels of the selected genes which comprise the WG score were utilized to calculate the WG Score for each patient in the TCGA cohort. The WG score was standardized by the Z method so that comparison between various cohorts and techniques could be performed.

### Network Analysis

The Agilent expression data for 130 patients was downloaded from TCGA database. The patients were divided into low risk and high risk patients based on SWG score. MeV software was used to perform the T-test. Genes with P value ≤0.05, after FDR correction were considered as significantly different. Genes having more than two fold difference between low risk and high risk were used for Gene Ontology (GO) and pathway analysis were carried out using DAVID bioinformatics resources and Kyoto Encyclopedia of Genes and Genomes (KEGG) pathways annotation and enrichment analysis respectively [25]–[27].

### Gene Expression Subtype Analysis

Gene expression subtype information, which was available for 108 patients out of 130 GBMs in the TCGA cohort was used for their correlation with survival. Patients from low risk and high risk groups were divided into gene expression subtypes and Mann Whitney test was carried out to find out significant association with the patient risk.

## Results

### Identification of a 14 Gene Prognostic Signature and Computation of Weighted Prognostic Gene (WG) Score

**Figure 1:**
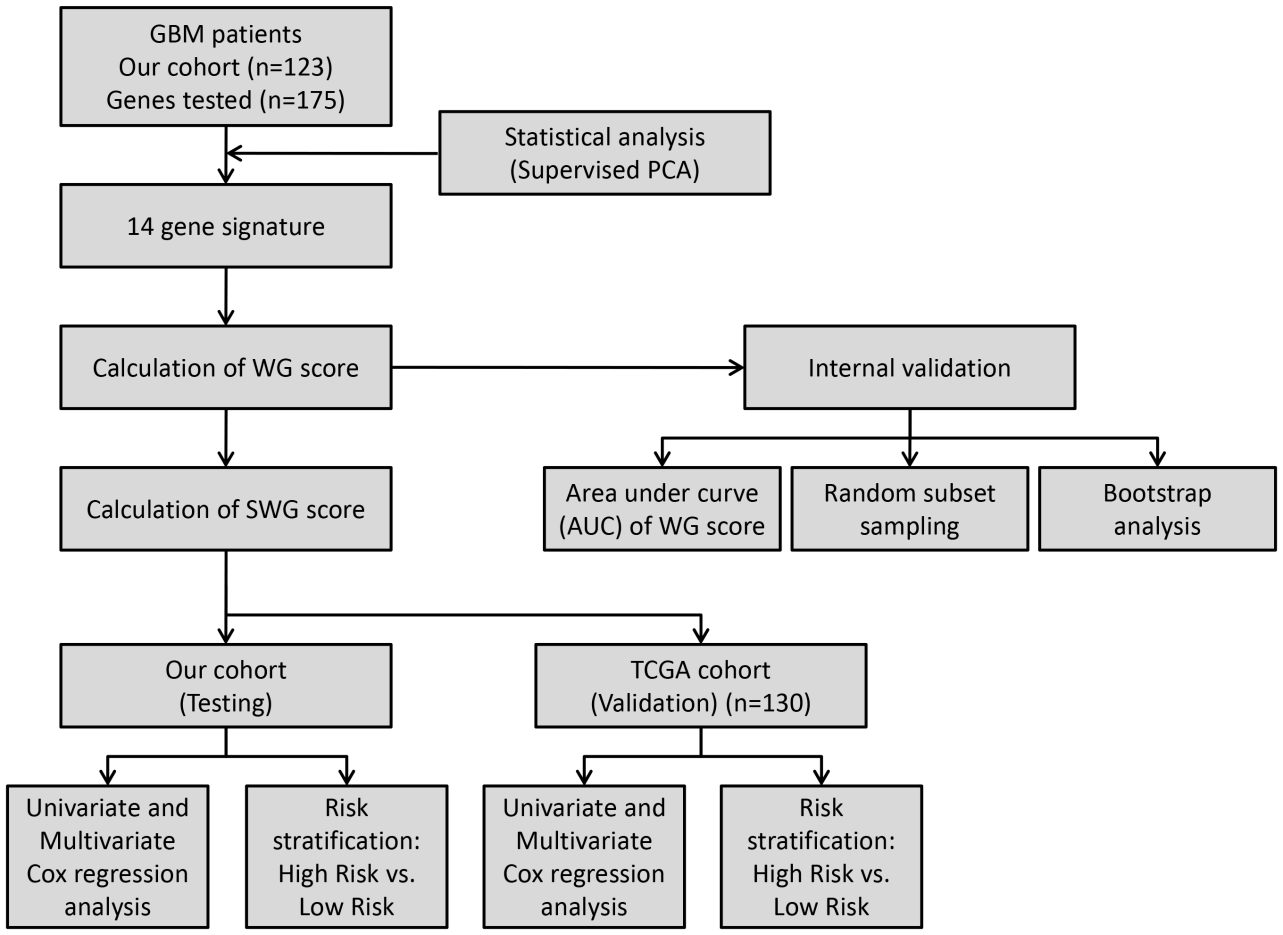
Schematic diagram describing the entire workflow as to how the 14 gene signature was identified, tested and validated is shown.

A schematic diagram describing the entire workflow as to how the 14 gene signature was identified, tested and validated is shown in **Figure 1**. The mRNA expression data of 175 genes was correlated with patient survival using a supervised principal component (SPC) method from the *superpc* software package in R with a Cox regression model. Genes were ranked by their univariate scores. The first principal component of the genes that reached a certain threshold of the univariate score was computed. An optimal threshold of 0^.^85 was selected based on likelihood ratio plot (**Supplementary Figure S1A and B**) to select the genes. On running a three component SPC model, the first supervised principal component was found to be significant (p = 0^.^0024), while the other 2 components were not. Genes were selected by the training and internal cross validation performed with the complete data set which finally yielded a group of 14 genes. This group of 14 genes provided a good approximation of the supervised principal component (**Table 1**).

**Table 1:** Details about the genes that form part of the 14 gene prognostic signature.

Sl No	Gene symbol	Gene name	Regressionco-efficient(Cox PH score)	Low risk			High risk		
				Median	Mean	SD*	Median	Mean	SD*
1	AGT	angiotensinogen	0.03190	−0.801	−2.335	4.366	0.1767	−0.290	2.319
2	EGFR	epidermal growth factor receptor	−0.05152	1.106	1.261	3.409	2.850	3.105	2.947
3	CHI3L1	chitinase 3−like 1	0.00442	3.600	3.782	2.965	6.934	6.168	2.624
4	SOD2	superoxide dismutase 2, mitochondrial	−0.08050	0**.**839	0.919	1.669	2.827	2.443	2.07
5	CCL2	chemokine (C-C motif) ligand 2	0.13690	−0.153	−0.574	1.911	1.560	1.219	2.037
6	IGFBPL1	Insulin-like growth factorbinding protein-like 1	−0.07953	2.413	2.586	3.168	−0.017	−0.236	2.576
7	MBP	myelin basic protein	0.07308	−6.881	−6.791	2.851	−5.010	−4.964	3.077
8	CPE	carboxypeptidase E	0.03070	−1.591	−2.224	2.462	−1.101	−1.241	1.258
9	OLFM1	olfactomedin 1	0.09206	−4.885	−4.837	2.446	−3.387	−3.736	1.769
10	MCF	MCF.2 cell line derivedtransforming sequence	−0.20973	−2.592	−1.954	2.528	−3.281	−3.569	2.475
11	PACSIN1	protein kinase C and caseinkinase substrate in neurons 1	0.01182	−6.411	−6.648	2.219	−6.085	−6.071	2.586
12	CALCRL	calcitonin receptor-like	−0.08132	2.569	2.300	1.802	1.438	1.204	2.353
13	SNCA	synuclein, alpha	0.17266	−4.633	−4.572	1.554	−3.588	−3.572	1.626
14	TOP2A	topoisomerase (DNA) II alpha	−0.12357	9.692	8.922	1.860	8.385	8.108	2.137

* SD - Standard deviation.

A weighted prognostic gene score (WG sore) (described in methods section) was calculated as a sum of Cox regression coefficients multiplied by the log2 ratio of each gene from the 14 gene signature. The WG score in the cohort ranged from −4^.^590 to −0^.^600, with the mean value of −2^.^414. The weighted prognostic gene score was found to significantly correlate with patient survival as a continuous variable (HR = 2^.^65; B = 0^.^975; p<0^.^001; Cox regression univariate analysis) (**Table 2** **)**.

**Table 2:** Univariate and multivariate analysis of impact of WG score on patient survival.

Factor	HR	B (co-efficient)	P value
**Present data set- survival analysis**			
**I. Univariate analysis**			
Age	1.025	0.025	0.006
WG score	2.65	0.975	<0.001
**II. Multivariate analysis**			
Age	1.016	0.016	0.092
WG score	2.507	0.919	<0.001
**TCGA data set- survival analysis**			
**I. Univariate analysis:**			
Age	1.028	0.027	<0.001
WG score	1.914	0.649	0.002
**II. Multivariate analysis:**			
Age	1.025	0.024	0.001
WG score	1.585	0.460	0.034

### Internal Validation of WG Score for Robustness

The robustness of WG score in predicting patient survival was assessed by multiple approaches. The fitness of the WG score, evaluated by calculating *A*rea *u*nder *R*OC Curve (AUC) with the time dependent censored survival data using Kaplan-Meier and span method, revealed that the area under the curve was 0^.^771 for WG score based on 14 genes (**Supplementary Figure S1C**) implying a higher predictive value of patients survival by the WG score. Secondly, the predictive value of WG score was analyzed in a subset of sixty two cases selected by random subset sampling. The WG score as a continuous variable was significant in predicting survival in this subset (p<0^.^0001). Patients were divided into two groups by mid-score, because of the expected differential proportion of patients in low risk and high risk groups. The mid score is the value which divides the range of WG score into equal parts. The<mid-score and ≥ mid-score groups also had significant difference between their median survival (p = 0^.^0002) (data not shown). Thirdly, a bootstrap internal validation was carried out to test the validity of the genes and WG score. The percentage of significant (P<0^.^05) prediction of survival duration by WG score was calculated from 1000 bootstrap samples of different sizes (n). The percentage significance was 69% for n = 10, 86% for n = 20, and 94% for n = 30 (**Supplementary Figure S1D**). This underscores the reproducibility of the WG score prediction even in smaller subsets of patients. We also assessed similar gene scores based on the linear regression on first principal component (**refer Supplementary Figure S1D, linear14 curve**) and the score based on minimal genes (**5 genes; refer Supplementary Figure S1D coxph5 curve**) selected by stepwise multivariate Cox survival model (data not shown). We noted that the 14 gene multivariate Cox survival model based WG score was better compared to scores derived from other methods (**Supplementary Figure S1D**).

### Multivariate Regression Analysis Indicates WG Score is an Independent Predictor of Patient Survival

Cox multivariate analysis was carried out using WG score, age and pre and post operative KPS as covariates. Pre operative KPS (p = 0.265) and post operative KPS (p = 0.549) did not significantly influence patient survival in the present cohort and hence not included for multivariate analysis. Age and WG score were both found to be significantly influencing the outcome on univariate analysis (**Table 2**). Multivariable analysis revealed that only WG score was found to significantly predict outcome (HR = 2^.^507; B = 0^.^919; p<0^.^001; **Table 2**). Hence, the WG score derived using 14 gene prognostic signature in this study is an independent predictor of survival outcome in this cohort of patients of GBM. This result also indicates that a higher WG score predicts poorer prognosis in patients with GBM.

### Independent Validation of 14 Gene Prognostic Signature using TCGA Data

**Figure 2:**
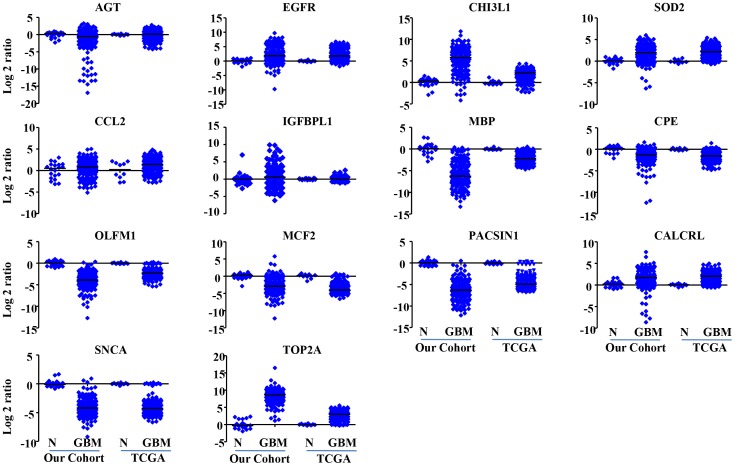
Scatter plots of 14 genes from the present study and TCGA study. Normal and GBM values are derived from RT-qPCR data for the present study. Log2-transformed gene expression ratios obtained from real-time RT-qPCR analysis of RNA derived from tumor tissue samples (as indicated) are plotted. For TCGA study derived data, the values are derived from microarray study performed using Agilent platform, downloaded from the TCGA data portal.

One hundred and thirty GBM patients from TCGA study who satisfied our criteria (see methods for details) were used for validation. It was observed that the expression patterns of all these 14 genes were similar both in our patient data set and the TCGA data set (**Figure 2** **and Supplementary Table S5**). The WG score calculated for TCGA data set ranged from −2^.^25 to −0^.^09 (Mean ± SD = −1^.^069±0^.^428). Univariate survival analysis identified both age and WG score as significant predictors of survival (**Table 2**). An increasing age as well as an increasing WG score predicted poorer outcome. Further, multivariate Cox proportional hazard model identified both age and WG score as independent predictors of survival (**Table 2**). Significantly, WG score demonstrated a higher hazard ratio than age in influencing the survival. We were also curious to carry out multivariate analysis with previously reported gene signatures: 4 gene signature [13] and 9 gene signature [10]. In univariate analysis, both the 14 gene and 9 gene signatures predicted survival significantly while the 4 gene signature reached nearing significance (**Supplementary table S6**). In a pairwise multivariate analysis which included 4 gene and 14 gene signatures, both signatures predicted survival significantly (**Supplementary table S6**). However, in another pairwise multivariate analysis which included 9 gene and 14 gene signatures, only 9 gene signature remained significant (**Supplementary table S6**).

### Risk Stratification by WG Score

Next we attempted to stratify the patients based on WG score to predict their survival. We found the range of WG score for the present study vs. TCGA cohort was different (**Supplementary Figure S2**) as different platforms (qRT-PCR and microarray respectively) were used in these two different data sets. Hence there was a need to standardize the WG score that is applicable to data obtained using various methods. Therefore, we standardized the WG score of our patient cohort and TCGA cohort by the Z statistic method. This method involves substituting all raw expression values in each data set by their respective *Z*-scores, which was calculated by (X − μ)/σ, where X stands for expression data of each gene in each of the sample; μ stands for mean of expression of each gene among all the samples; and σ stands for standard deviation. This yielded a standardized WG score (SWG score). The SWG score of the present study cohort ranged from −2^.^795 to 2^.^330, while the SWG score of the TCGA cohort ranged from −2^.^770 to 2^.^276. The mid value of the SWG score for the present study cohort was calculated to be −0^.^2327, which was utilized as cut off for risk stratification (SWG rounded off to −0^.^233) in both the cohorts. All patients with SWG score of ≤−0^.^233 were classified as low risk group, while those with SWG score>−0^.^233 were classified as high risk group.

**Figure 3:**
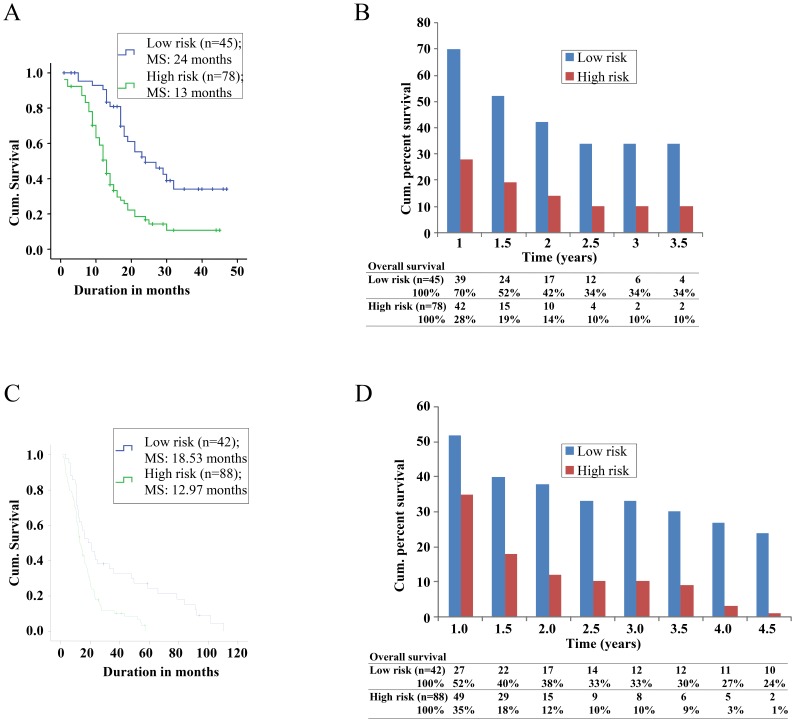
Risk stratification of GBM patients. Survival by risk stratification of patients from present study group (**A** and **B**) and TCGA cohort (**C** and **D**).Kaplan-Meier graph showing overall survival of GBM patients for the present cohort (**A**) and TCGA cohort (**C**) according to standardized WG score. Comparison of overall survival rates of low risk group vs. high risk group throughout the study period of present study group (**B**) and TCGA cohort (**D**).

**Figure 4:**
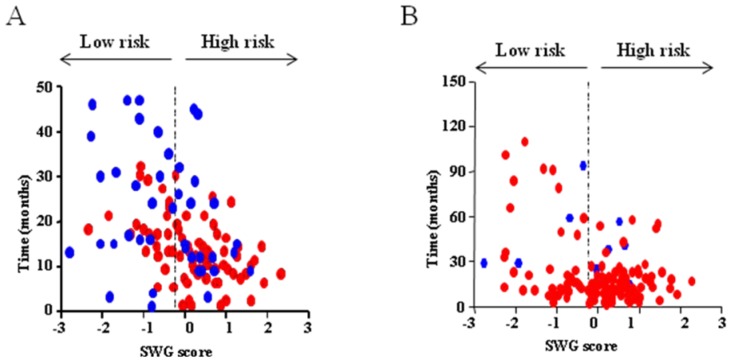
Comparison between patient’s survival and WG risk score. Comparison of patient survival status with WG scores for the present study group (**A**) and the TCGA cohort (**B**). Patients who are alive or dead are indicated by blue or red dots respectively.

It was noted that the median survival of patients in low risk group was significantly higher than that of the high risk group both in the present and in the TCGA data sets by the Kaplan Meier method (**Supplementary Table S7**; **Figure 3A and C**). To get a visual appreciation, a comparison of SWG score with patient survival status among GBM patients between low and high risk groups of the present data set and the TCGA data set are shown in **figure 4A and B**. The presence of more red dots (dead patients) in the high risk group in both the data sets is evident. The survival rates of the low risk and high risk groups revealed that the low risk group had better survival rates throughout the study period in both the present study as well as TCGA cohort compared to the high risk group (**Figure 3B and D**). A multivariate analysis demonstrated that the stratification of patient cohort into low and high risk remained significant in both the present study cohort and the TCGA cohort, after correcting for the influence of patient’s age (**Table 3**).

**Table 3:** Multivariate analysis of risk stratification by SWG score.

Factor	HR	B Coefficient	P value
I - Present data set			
Age	1.018	0.018	0.051
SWG score (high vs low)	2.595	0.953	<0.001
II - TCGA data set			
Age	1.023	0.023	0.002
SWG score (high vs low)	1.710	0.536	0.017

### Identification of a Patient Cohort with very Low Risk using SWG Score

**Figure 5:**
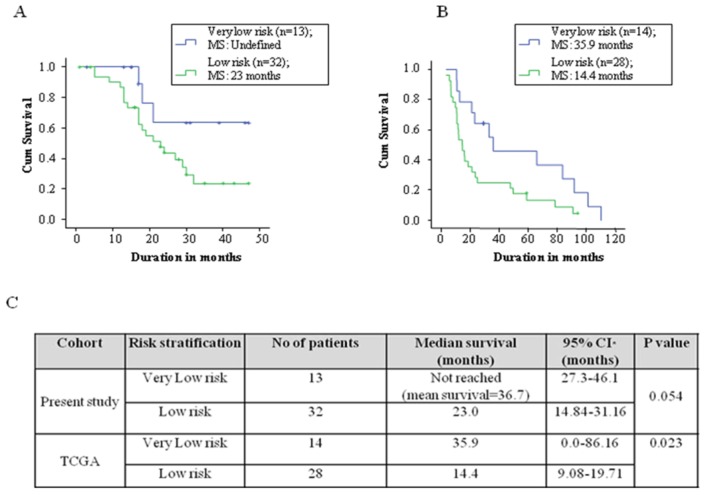
Risk stratification of low risk GBM patients. Survival by risk stratification of patients from present study group (**A**) and TCGA cohort (**B**). Kaplan-Meier graph showing overall survival of glioblastoma patients that belong to low risk (as identified Figure 1) into very low risk and low risk for the present cohort (**A**) and TCGA cohort (**C**) according to standardized WG score**.** Please note that the low risk group (as identified from Figure 1) was further divided into low risk and very low risk by 30^th^ percentile value of the SWG score, which identified a small cohort of patients with very long median survival.

Patients with low risk were further divided based on the standardized weighted gene (SWG) score. The 30^th^ percentile of the range of SWG score of the present study cohort was taken as the cut off, namely a value of −1.258. All patients with a SWG score of ≤ −1.258 were grouped as very low risk, while those with a SWG score between −1.258 and −0.233 were grouped as low risk. It was noted that the very low risk group in our cohort had a very long survival (median survival not reached; mean survival = 36.7 months) while compared to the low risk group (median survival = 23 months) (p = 0.054). Similarly, it was noted that the very low risk group in TCGA had a significantly higher median survival compared to low risk group (35.9 vs 14.4 months respectively) (p = 0.023) (**Figure 5A, B and C**).

### Nature of Genes Involved in SWG Score Computation

Several interesting observations were noted when we had a closer look at the 14 genes that formed the signature. It was interesting to note that among the 14 genes which comprised the SWG score, some had known significant functions in the glioma pathogenesis or malignant potential, while some were novel (**Table 1** **)**. Considering the expression of 14 genes, four genes (IGFBPL1, MCF2, CALCRL, and TOP2A) were expressed at a higher level in the low risk group compared to the high risk group, probably having protective effect, while the remaining 10 genes (SOD2, EGFR, AGT, CHI3L1, CCL2, MBP, CPE, OLFM1, PACSIN1, and SNCA) were expressed at a higher level in the high risk group than in the low risk group, probably having a role in tumor aggressiveness (**Table 1**). Similar results were obtained in the TCGA cohort as well (**Supplementary Table S8**).

### Analysis of Protein Expression

**Figure 6:**
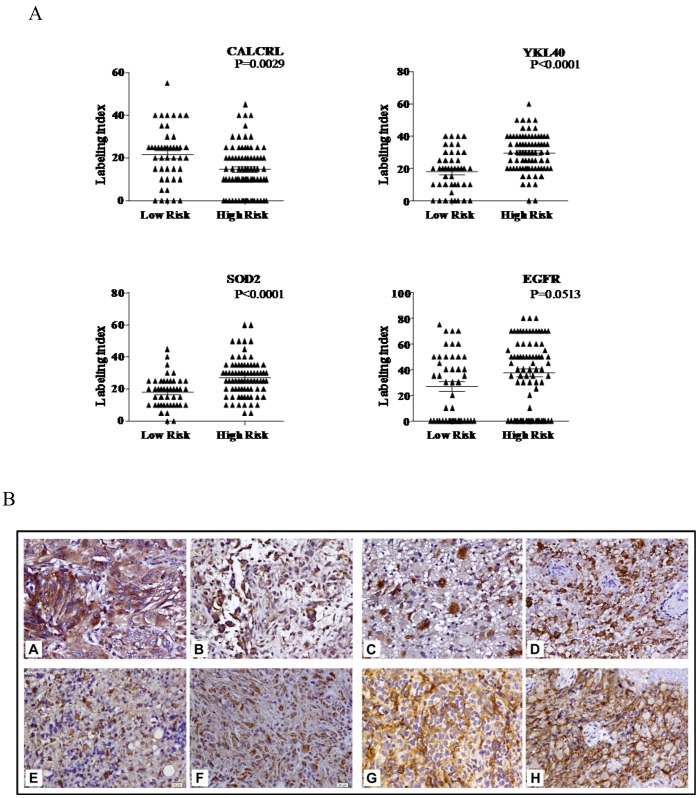
Immunohistochemical analysis of selected genes. Immunohistochemical staining pattern of the proteins of CALCRL that was expressed at a higher level in the low risk groups and CHI3L1, SOD2 and EGFR that were expressed at a higher level in the high risk groups of glioblastoma. **A**) Labeling index of these four proteins between low risk and high risk patients is shown. **B**) CALCRL (A, B) CHI3L1 (C, D) and SOD2(E, F) show cytoplasmic staining; EGFR (G, H) shows membrane staining of tumor cells. All original magnifications are ×160.

The protein expression of four of the genes, namely EGFR, CHI3L1, SOD2 and CALCRL was performed on 123 cases (from our cohort) to correlate with transcript levels. Spearman’s correlation analysis revealed that the protein levels (labeling index by IHC) correlated significantly with mRNA levels for all the four genes, namely EGFR (p<0.001; correlation co-efficient = 0.488),CHI3L1 (p<0.001; correlation co efficient = 0.601), SOD 2 (p<0.001;correlation co-efficient = 0.730) and CALCRL (p<0.001;correlation co-efficient = 0.757). It is evident that while CALCRL, a protective gene, is found to be significantly expressed higher in low risk group compared to high risk group (**Figure 6** **A and B**), the risky genes were found to be expressed significantly higher in high risk group compared to low risk group (**Figure 6** **A and B**) except EGFR, which showed nearing significance (p = 0.0513.

### Pathway Analysis Reveals Active Inflammatory Response Pathway in High Risk Group

We hypothesized that the 14 genes, which formed the signature, might modulate global gene expression with the resultant perturbation of key signaling pathways differently between low risk and high risk patients which could possibly explain the difference in the survival between these two groups. Gene expression profiles from Agilent data set from TCGA for low risk and high risk groups were compared by statistical analysis. There were 76 genes identified which are significantly differentially regulated (73 genes upregulated and 3 genes were down regulated in high risk compared to low risk) with more than two fold difference between these two groups (**Supplementary Table S9**). These 76 genes were used as input for the GO and pathway analyses.

**Figure 7:**
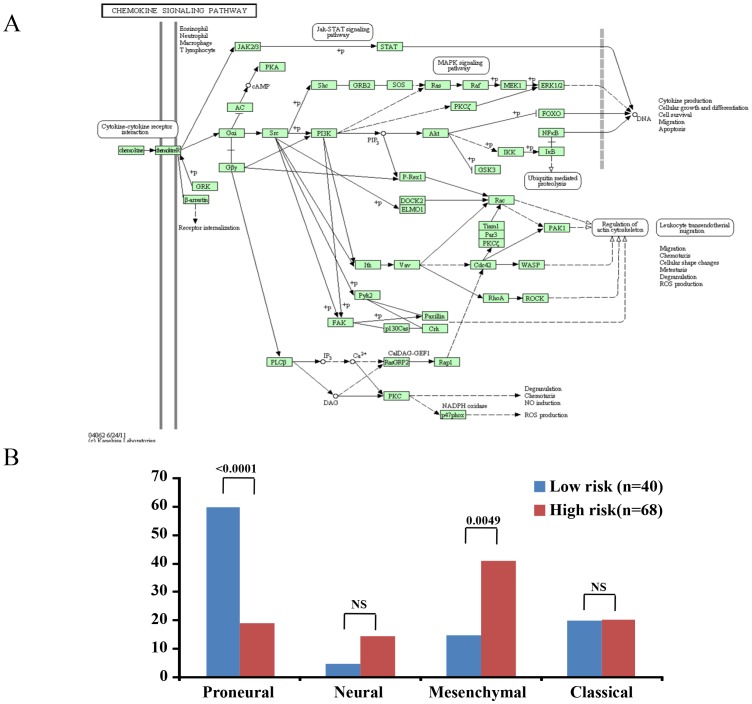
Pathway analysis and gene expression subtype analysis. **A**) KEGG pathway enrichment analysis. Enrichment of cytokine-cytokine receptor interaction and chemokine signaling pathways leads to activation of various pro-survival pathways like Jak-STAT pathway, MAPK signaling pathway and NFkB pathway with the resultant pro-tumorigenic environment. **B**) TCGA patients (n = 108) were divided into gene expression sub types – proneural, neural, mesenchymal and classical among low risk and high risk groups. Mann Whitney test was carried out to find out significance of distribution of a given expression subtype within a risk group.

GO analysis revealed that these 76 genes were enriched in 16 biological processes (p<0.05), most of which were related to inflammatory and immune response (**Table 4**). Further, KEGG (Kyoto Encyclopedia of Genes and Genomes) pathway analysis identified these differentially expressed genes annotated into three different pathways namely Cytokine-cytokine receptor interaction (hsa04060;11/76 genes; OSM, CXCL1, TSLP, CCL2, CXCL14, IL8, CCL20, CXCR4, CXCL3, IL18 and FAS; p = 3.14E-05), NOD-like receptor signaling pathway (hsa04621;6/76 genes; CXCL1, CCL2, IL8, IL18, CASP1 and BIRC3; p = 4.44E-04), and Chemokine signaling pathway (hsa04062; 8/76; CXCL1, CCL2, CXCL14, IL8, CCL20, CXCR4, CXCL3 and GNG4; p = 0.0024 (**Figure 7A**). These results suggest that activation of inflammatory and immune response processes might contribute to the shorter survival of high risk patients.

**Table 4:** Gene Ontology terms significantly enriched (p<0.05) in the set of genes differentially expressed between high risk and low risk.

Sl. No.	Biological process	GO term	No. of genes	P value
1	Inflammatory response	GO:0006954	18	1.00E-10
2	Response to wounding	GO:0009611	20	1.60E-09
3	Defense response	GO:0006952	20	2.10E-08
4	Taxis	GO:0042330	12	1.53E-07
5	Chemotaxis	GO:0006935	12	1.53E-07
6	Immune response	GO:0006955	18	9.25E-06
7	Locomotory behavior	GO:0007626	12	4.39E-05
8	Behavior	GO:0007610	14	1.79E-04
9	Regulation of response to external stimulus	GO:0032101	9	6.43E-04
10	Leukocyte chemotaxis	GO:0030595	6	6.81E-04
11	Cell chemotaxis	GO:0060326	6	8.93E-04
12	Neutrophil chemotaxis	GO:0030593	5	1.17E-03
13	Leukocyte migration	GO:0050900	6	6.08E-03
14	Chemical homeostasis	GO:0048878	12	1.97E-02
15	Homeostatic process	GO:0042592	14	3.15E-02
16	Cell migration	GO:0016477	9	3.67E-02

Various molecular subtypes of GBM have been previously described by Phillips et al. to predict tumor biology [28]. We analyzed their distribution between the low and high risk group stratification based on the 14 gene signature, to understand the possible correlation of immune pathway with molecular subgroups. We found that high risk group had significantly higher proportion of mesenchymal subtype while the low risk group had significantly higher proportion of proneural subtype (**Figure 7B**). Subsequently, we checked whether protective and risky genes are up regulated in proneural and mesenchymal sub groups respectively. We found that all four protective genes (IGFBPL1, MCF2, CALCRL, and TOP2A) are up regulated significantly in proneural group compared to mesenchymal group (**Supplementary table S10**). Among the 10 risky genes, seven genes (SOD2, EGFR, CHI3L1, CCL2, MBP, CPE, and SNCA) were found to be up regulated with three genes showing significant association in mesenchymal group compared to proneural group (**Supplementary table S10**). This result suggests that the presence of activated inflammatory/immune response pathway in the high risk group may be related to enriched mesenchymal subtype.

## Discussion

GBM is characterized by its heterogeneity at its cellular, molecular, and genetic levels, which render them one of the most complex groups of tumors being studied. As the genetic basis of origin and progression of these treatment resistant tumors becomes amply clear by multiple studies, newer molecules, and areas of targets for therapy and prognostication are continuously being sought after. Recent research has been focused on identification of gene signatures which can predict prognosis in glioma [10], [29], [30]. Various statistical methods including unsupervised analysis [29], [31], integrated genomic analysis [30], and systems biology approach [32] have been employed in these studies. Most of the studies which included malignant glioma or GBM contained retrospective patient cohort from various trials or centers and hence had not been treated with uniform therapy [17], [33], [34]. Since the survival of GBM patients also depends on the type of adjuvant therapy, non uniform therapy can have possible confounding effects on the analysis [1], [3].

The strengths of the present study include a homogenous group of patients of GBM, prospectively recruited based on strict inclusion criteria and treated with standard adjuvant therapy. This cohort was a fit model to evaluate the prognostic impact of genes on survival in a homogenous histological group namely GBM. In the present study, clinical factors like KPS did not correlate with the survival. Age was the only clinical factor which correlated with survival and hence the WG score generated from the expression of genes was evaluated for its impact on survival adjusting for the significance of age on survival.

We identified a 14 gene prognostic signature by supervised principal component analysis, which significantly correlated with survival independent of the effects of age. Since our cohort had patients with GBM, who underwent standardized treatment protocol and selected with other stringent criteria, we attempted to evaluate the utility of the 14 gene prognostic signature in other cohorts where the patients did not receive uniform therapy. Such a cohort was available from the publicly available TCGA consortium. We demonstrated that the 14 gene signature significantly correlated with survival in the TCGA data also. Furthermore, we were also able to divide the low risk group further to identify a very low risk group based on 30^th^ percentile, which had a significantly better survival.

The scrutiny of functional significance of the 14 genes suggested a differential pattern of gene expression between the low and high risk groups. Investigation of known functions of many of these genes justified the association between their expression and patient survival. Among the genes overexpressed in low risk group, Insulin-like growth factor binding protein-like 1 (IGFBPL1) was originally identified as a novel putative tumor suppressor protein which was later found to be a hypermethylated gene in primary breast cancers that was associated with disease free survival [35]. MCF-2 encodes a guanine nucleotide exchange factor (GEF) that activates the rho family of GTPases. Interestingly, a variant of this gene with a deletion of 10 amino acids at exon10/11 with a deficient GEF activity that was found overexpressed in normal brain is downregulated in GBMs [36]. TOP2A, which encodes topoisomerase II alpha is associated with cellular proliferation but the overexpression is associated with better response upon treatment with TOP2A inhibitors [37]. We have recently demonstrated that temozolomide is a TOP2A inhibitor thus explaining the phenomenon of TOP2A overexpressing tumors being more sensitive to temozolomide chemotherapy with resultant longer survival of patients. Further, down regulation of TOP2A using RNA interference in glioma cells resulted in temozolomide chemosresistance [38]. Unlike these three genes, CALCRL (***Calc***itonin ***r***eceptor-***l***ike), has been shown to activate angiogenesis and was found to be expressed in endothelial cells of microvascular proliferations and in the neighbouring tumor cells of glial tumors [39], [40].

Among the genes overexpressed in high risk group, EGFR has known oncogenic functions such as cell migration, survival, angiogenesis, and tumorigenesis [41]. Similarly, YKL-40, a member of mammalian chitinase-like proteins has been found to be associated with poor prognosis in many cancers including glioma [42]. CCL2/MCP1 has been shown to be protumorigenic and a target for therapy in glioma and other cancers [43]. MBP has been shown to induce cellular proliferation, inhibit apoptosis, and found to be associated with advanced stages of cancer and poor prognosis [44]. SNCA (Synuclein, alpha), an abundantly expressed protein in brain with a possible involvement in presynaptic signaling and membrane trafficking, has been shown to inhibit apoptosis by inhibiting caspase 3 thus justifying its identification as a poor prognostic indicator [45]. There is no information available regarding the other genes; CPE, OLFM1, and PACSIN1 with respect to their function (s) in tumor biology, whose expression is higher in high risk group, GO and KEGG pathway analyses revealed significant enrichment of cytokine-cytokine receptor interaction and chemokine signaling pathway which are related to inflammatory and immune response pathways. While acute inflammation is related to innate and humoral immunity, leading to immune surveillance with the resultant anti-tumor effect, chronic inflammation has pro-tumorigenic effect supporting tumor initiation and progression [46], [47]. Activated oncogenes have been shown to induce various chemokines and cytokines resulting in the recruitment of inflammatory cells in the tumor milieu [48]. The recruited inflammatory cells themselves secrete more chemokines and cytokines resulting in the generation of cancer-related inflammation which supports tumor growth and progression [48]. In fact, pathway analysis identified the enrichment of cytokine-cytokine receptor interaction pathway and chemokine signaling pathway specifically in the high risk group. An active inflammatory environment produced by these two pathways can lead to reduced apoptosis, increased proliferation, migration and subsequently metastasis. Thus the specific activation of inflammatory and immune response pathway in the high risk group of patients might explain their shorter survival.

Although different gene expression subtypes in GBMs appears to have similar patient survival, it has been shown recently that there is an enrichment of immune response-related gene expression in the mesenchymal subtype of adult GBM [30], [49]. Further it has been demonstrated that there is a role for immune response, particularly the microglia/macrophage response, in the biology of mesenchymal subset of GBM with poor prognosis [49]. Hence it was of our interest to see whether the high risk group (as identified by 14 gene expression signature) with activated inflammatory/immune response pathways is enriched for mesenchymal subtype. Interestingly, we noted that the high risk group which had enrichment of activated inflammatory/immune response pathway also had higher occurrence of mesenchymal subtypes in GBMs, raising an interesting possibility of an influence of immune pathways in the mesenchymal subtype of GBM.

A number of gene expression profiling platforms like Affymetrix [10], [31], Agilent [24], cDNAarrays, RNA expression by real time PCR have been used in various studies for the measurement of gene expression in gliomas. Keeping in mind that the gene expression values derived from various platforms may not be directly comparable, we utilized the standardized values for correlation and risk stratification. It was noted that stratification into high and low risk groups developed based on the SWG score predicted survival significantly in our set (obtained by real time PCR) and TCGA cohort (obtained by Agilent microarrays). This observation gains significance since the SWG score can be applied to gene expression analysis by any platform after standardizing the WG score.

Most of the previous studies which reported gene signatures have demonstrated the requirement of large number of genes or gene clusters for forming a gene signature [29], [30]. For example, Colman *et al* have reported a 38 gene and 9 gene prognostic signature model based on the data from 4 previously reported studies [10]. deTayrac *et al* reported a 4 gene signature which predicted survival in malignant glioma [13]. In the present study, the derivation of the SWG score by a simple weightage based formula and standardization to any platform obviates the need for any complex computational algorithms or gene cluster approaches in reproducing the analysis and utilizing the score in patient stratification.

We compared the genes comprising the SWG score to previously reported studies. Only a few studies have analyzed the utility of gene signatures in GBM prognosis. It was interesting to note that the genes comprising our gene signature were largely unique and independent from the previously reported signatures [32], [33]. CHI3L1 was the only gene which figured in both our signature and that reported by Colman *et al* [10]. Our set of genes were unique and different from the four genes namely, CHAF1B, PDLIM4, EDNRB, and HJURP, which were identified in the study by de Tayrac *et al* [13].

The identification of gene signature with an ability to stratify the GBM patients into low and high risk groups based on an easily derivable SWG score would enable the administration of individualized therapy. Patients with high risk can be managed with more aggressive and multimodal adjuvant therapy. In conclusion, we report a 14 gene signature whose expression correlates with the patient survival out come in GBM which could also be used to stratify patients into low and high risk groups.

## Supporting Information

Figure S1 **Identification and validation of fourteen gene signature.**
(PPT)

Figure S2 **Range of WG score.**
(PPT)

Table S1 **Details of primers used for RT-qPCR.**
(DOCX)

Table S2 **List of genes selected for expression analysis by RT-qPCR.**
(DOCX)

Table S3 **Case bias analysis in the present data set.**
(DOCX)

Table S4 **Genes selected from full data during cross validation.**
(DOCX)

Table S5 **Comparison of gene expression in present data set and TCGA data set.**
(DOCX)

Table S6 **Univariate and multivariate analysis of WG score with other signatures.**
(DOCX)

Table S7 **Survival based on risk stratification by SWG score.**
(DOCX)

Table S8 **The expression pattern of genes in low risk and high risk groups in the TCGA data set.**
(DOCX)

Table S9 **List of differentially regulated genes between high risk and low risk patients derived from TCGA data set.**
(DOCX)

Table S10 **Transcript levels of 14 genes in four glioma subtypes.**
(DOCX)
