# Supplementary material for: A Fourteen Gene GBM Prognostic Signature Identifies Association of Immune Response Pathway and Mesenchymal Subtype with High Risk Group
Source: PLoS One. 2013 Apr 30;8(4):e62042. doi: 10.1371/journal.pone.0062042 (PMC3639942; doi:10.1371/journal.pone.0062042)
Supplement: Figure S1 — Identification and validation of fourteen gene signature. (PPT) [file pone.0062042.s001.ppt]

## Slide 1
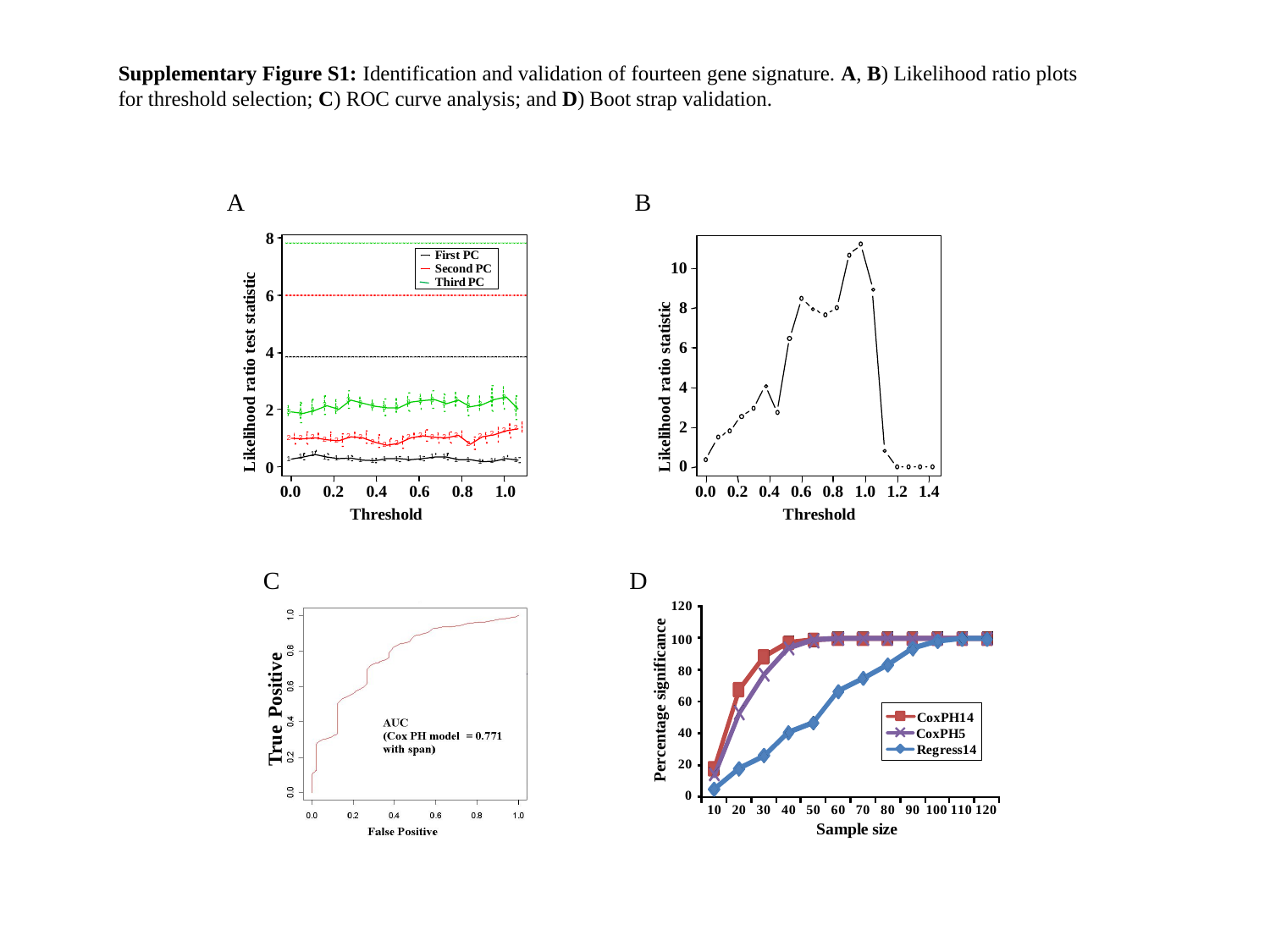

Supplementary Figure S1: Identification and validation of fourteen gene signature. A, B) Likelihood ratio plots for threshold selection; C) ROC curve analysis; and D) Boot strap validation.
