# Supplementary material for: A Fourteen Gene GBM Prognostic Signature Identifies Association of Immune Response Pathway and Mesenchymal Subtype with High Risk Group
Source: PLoS One. 2013 Apr 30;8(4):e62042. doi: 10.1371/journal.pone.0062042 (PMC3639942; doi:10.1371/journal.pone.0062042)
Supplement: Figure S2 — Range of WG score. (PPT) [file pone.0062042.s002.ppt]

## Slide 1
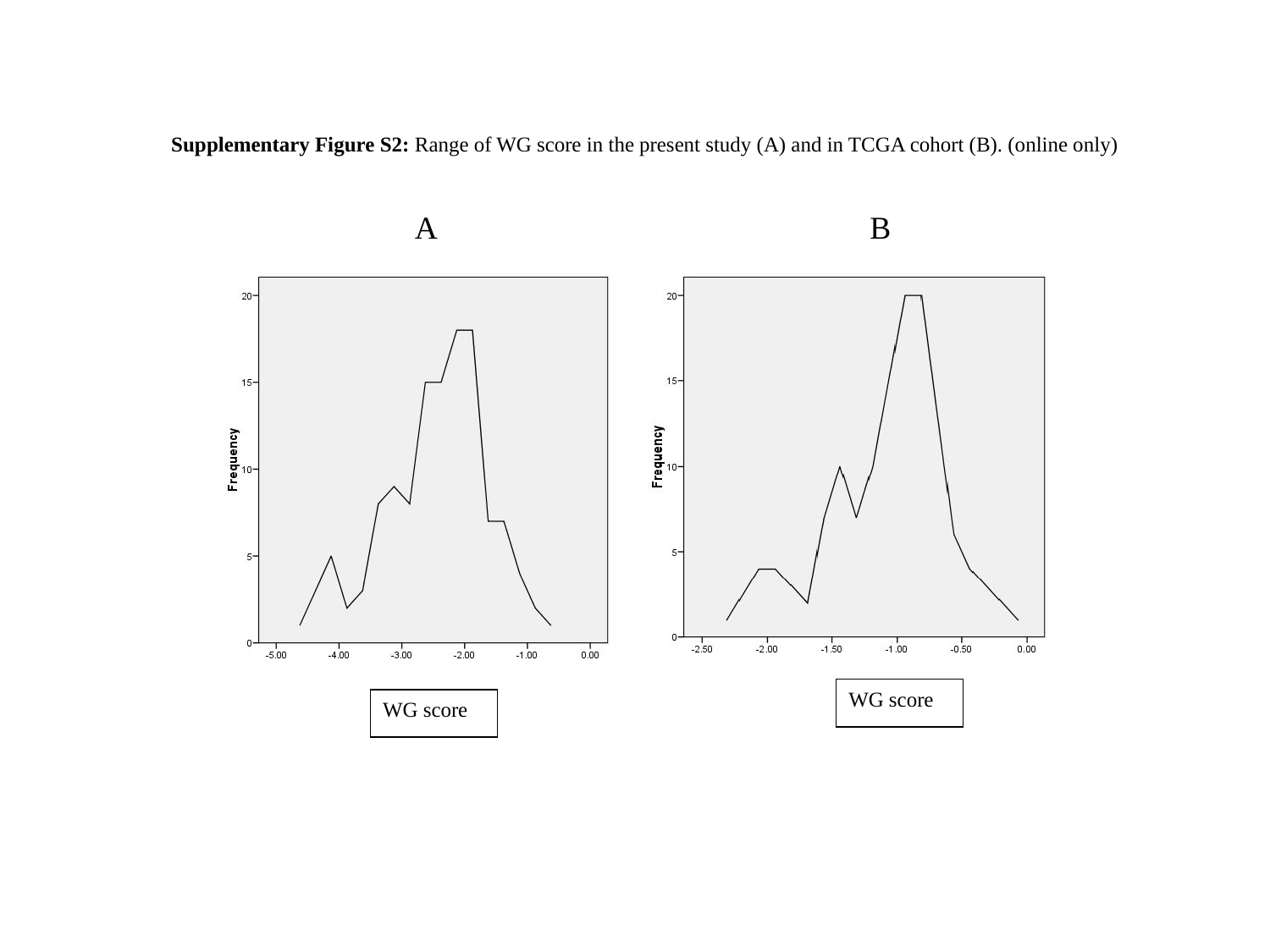

Supplementary Figure S2: Range of WG score in the present study (A) and in TCGA cohort (B). (online only)
A
B
WG score
WG score
