# Supplementary material for: A Fourteen Gene GBM Prognostic Signature Identifies Association of Immune Response Pathway and Mesenchymal Subtype with High Risk Group
Source: PLoS One. 2013 Apr 30;8(4):e62042. doi: 10.1371/journal.pone.0062042 (PMC3639942; doi:10.1371/journal.pone.0062042)
Supplement: Table S1 — Details of primers used for RT-qPCR. (DOCX) [file pone.0062042.s003.docx]

**Supplementary table S1**: Details of primers used for RT-qPCR

| **GENE NAME** | **GENE FULL NAME** | **F/R^*^** | **Sequence ( 5' --> 3' )** |
| --- | --- | --- | --- |
| 18S | ribosomal RNA | F | TAACAGGTCTGTGATGCCCT |
| 18S | ribosomal RNA | R | TCAAGTTCGACCGTCTTCTC |
| ADD3 | adducin 3 | F | GCCAAGGCGTGATTACCACT |
| ADD3 | adducin 3 | R | TGCAGGATCTGAGTAACTCGTTT |
| AEBP1 | AE binding protein 1 | F | TGCTCTGTGGCCCCTGTCTACA |
| AEBP1 | AE binding protein 1 | R | CACCTTCATGAGCTGGCGCAT |
| AGPAT1 | acylglycerol-3-phosphate O-acyltransferase | F | GACGCAACGTCGAGAACATGAA |
| AGPAT1 | acylglycerol-3-phosphate O-acyltransferase | R | CTCCATCATCCCAAGCAGATCG |
| AGT | angiotensinogen | F | CCCCAGTCTGAGATGGCTC |
| AGT | angiotensinogen | R | GAGGTGGAAGGGGTGTATGT |
| AQP1 | aquaporin 1 (channel-forming integral protein, 28kDa) | F | TTAACCCTGCTCGGTCCTTTG |
| AQP1 | aquaporin 1 (channel-forming integral protein, 28kDa) | R | AGTCGTAGATGAGTACAGCCAG |
| ARC | activity-regulated cytoskeleton-associated protein | F | CTGTCCCAGATCCAGAATCACATGA |
| ARC | activity-regulated cytoskeleton-associated protein | R | TTGCGCCACAGGAACTGGTC |
| ARHC | ras homolog gene family, member C | F | CCTGCCTCCTCATCGTCTTC |
| ARHC | ras homolog gene family, member C | R | AGCACATGAGGATGACATCAGTG |
| ASCL1 | achaete-scute complex homolog 1 | F | AAGCTCTGCCAAGATGGAGA |
| ASCL1 | achaete-scute complex homolog 1 | R | GGCAAAGAAACAGGCTGC |
| ATP5G1 | ATP synthase, H+ transporting, mitochondrial F0 complex, subunit C1 | F | CCAGACGGGAGTTCCAGAC |
| ATP5G1 | ATP synthase, H+ transporting, mitochondrial F0 complex, subunit C1 | R | GACGGGTTCCTGGCATAGC |
| B2M | beta-2-microglobulin | F | AGGCTATCCAGCGTACTCCAA |
| B2M | beta-2-microglobulin | R | AATGCGGCATCTTCAAACC |
| BCAN | brevican | F | GCCTGACAGCTACTTCCTGTCTGGA |
| BCAN | brevican | R | CAGGACACCAGCCCCATCTTG |
| BHC80 | PHD finger protein 21A | F | AAAGTGGAAATTCAGGTTCACCA |
| BHC80 | PHD finger protein 21A | R | TCAGGTTCTTCCGTAGCTGTT |
| BMP2 | bone morphogenetic protein 2 | F | ACCCGCTGTCTTCTAGCGT |
| BMP2 | bone morphogenetic protein 2 | R | CTCAGGACCTCGTCAGAGGG |
| C1QB | complement component 1, q subcomponent, B chain | F | AGGGAACCTGTGCGTGAAC |
| C1QB | complement component 1, q subcomponent, B chain | R | CTCCATGCCCAGTAGTGAGTT |
| C5orf18 | polyposis locus protein 1; deleted in polyposis 1; polyposis coli region hypothetical protein DP1 | F | GGCTGACCTACTGGGTAGTGT |
| C5orf18 | polyposis locus protein 1; deleted in polyposis 1; polyposis coli region hypothetical protein DP1 | R | GGATGATGCGCTTGTAGAGC |
| C6ORF66 | NADH dehydrogenase (ubiquinone) 1 alpha subcomplex, assembly factor 4 | F | AGCGGAACGGGAAATCAGC |
| C6ORF66 | NADH dehydrogenase (ubiquinone) 1 alpha subcomplex, assembly factor 4 | R | GCAGCTTTTACCTGCAAGGAA |
| CACNG4 | calcium channel, voltage-dependent, gamma subunit 4 | F | CGAAGGGATCTATAAAGGGCAC |
| CACNG4 | calcium channel, voltage-dependent, gamma subunit 4 | R | ACTGAGGACGATGTTGTTCTTG |
| CALCRL | calcitonin receptor-like | F | CAGCAAGCAACAGAACATGGA |
| CALCRL | calcitonin receptor-like | R | GTGATGCAATAGACAATCCGTGT |
| CBX3 | chromobox homolog 1 | F | TAGATCGACGTGTAGTGAATGGG |
| CBX3 | chromobox homolog 1 | R | TGTCTGTGGCACCAATTATTCTT |
| CCL2 | chemokine (C-C motif) ligand 2 | F | CGCTCAGCCAGATGCAATCAAT |
| CCL2 | chemokine (C-C motif) ligand 2 | R | GCTGGTGATTCTTCTATAGCTCGCG |
| CD99L2 | CD99 molecule-like 2 | F | TGGTAGCAAGAGTGACCGATGTC |
| CD99L2 | CD99 molecule-like 2 | R | CAAGCGAATGTCTCTTTCCCCTC |
| CDC2 | cell division cycle 2, G1 to S and G2 to M | F | AGGTCAAGTGGTAGCCATGAA |
| CDC2 | cell division cycle 2, G1 to S and G2 to M | R | ACAAAACACAATCCCCTGTAGG |
| CDKN3 | cyclin-dependent kinase inhibitor 3 | F | TCAGTTTCTCGGTTTATGTGCTC |
| CDKN3 | cyclin-dependent kinase inhibitor 3 | R | TTTTGACAGTTCCCCTCTGGT |
| CENPF | centromere protein F | F | ACAACTGTCACTTGAGCTGGA |
| CENPF | centromere protein F | R | GTGGGTTTCAATGCACCAGTC |
| CHI3L1 | chitinase 3-like 1 | F | GTGAAGGCGTCTCAAACAGG |
| CHI3L1 | chitinase 3-like 1 | R | TCAGGCTGGCACCTTTAGTG |
| CITED1 | Cbp/p300-interacting transactivator, with Glu/Asp-rich carboxy-terminal domain, 1 | F | GACCCCTGCAAAACTGGGAC |
| CITED1 | Cbp/p300-interacting transactivator, with Glu/Asp-rich carboxy-terminal domain, 1 | R | ACCAGCGACATCAGCACTTC |
| CLU | clusterin | F | ACGAAGAGCGCAAGACACTG |
| CLU | clusterin | R | ATGGTCTCATTGCACACTCCT |
| CNR1 | cannabinoid receptor 1 | F | AAGGTGACATGGCATCCAAAT |
| CNR1 | cannabinoid receptor 1 | R | AGGACGAGAGAGACTTGTTGTAA |
| COL4A2 | collagen, type IV, alpha 2 | F | TTGGCGGGTGTGAAGAAGTTT |
| COL4A2 | collagen, type IV, alpha 2 | R | CCTTGTCTCCTTTACGTCCCTG |
| COL6A1 | collagen, type VI, alpha 1 | F | ACACCGACTGCGCTATCAAG |
| COL6A1 | collagen, type VI, alpha 1 | R | CACCGAGAAGACTTTGACGC |
| COL6A3 | collagen, type VI, alpha 3 | F | TGGTCCAGTTCAACGGAAACC |
| COL6A3 | collagen, type VI, alpha 3 | R | CACTACGATAACCTGAGGGACT |
| CPE | carboxypeptidase E | F | TGCAACGAATACCAGAAGGGG |
| CPE | carboxypeptidase E | R | CCAGTCCTTGAGTTCACCAGG |
| CRB1 | crumbs homolog 1 | F | GCAGATGACTTGATCTCCGAC |
| CRB1 | crumbs homolog 1 | R | CCCTGAGTTGCCCTTTTGTTG |
| CRTAC1 | cartilage acidic protein 1 | F | CTGGCGCTCAGAGATGTGG |
| CRTAC1 | cartilage acidic protein 1 | R | CCATTCTCATTGTCGCAGAAGAT |
| CRYAB | crystallin, alpha B | F | GTTCTTCGGAGAGCACCTGTT |
| CRYAB | crystallin, alpha B | R | GAGAGTCCAGTGTCAAACCAG |
| CSDC2 | cold shock domain containing C2, RNA binding | F | AAGCGGACCAGGACCTATTCA |
| CSDC2 | cold shock domain containing C2, RNA binding | R | CCCCTCGATGTCAGACACA |
| CX43 | connexin 43 | F | TGTCCTTAAGTCCCTGCTAA |
| CX43 | connexin 43 | R | GTAGCTGAGGAATGATGAAAAAG |
| DBI | diazepam binding inhibitor | F | TTAAGACCAAGCCATCGGATG |
| DBI | diazepam binding inhibitor | R | CCTTTCAGCTCATTCCAGGC |
| DCN | decorin | F | AGTTGGAACGACTTTATCTGTCC |
| DCN | decorin | R | GTGCCCAGTTCTATGACAATCA |
| DCX | doublecortin | F | TGGAGATGCTAACCTTGGGTA |
| DCX | doublecortin | R | TCACACATGCCCACATGACTA |
| DDR1 | discoidin domain receptor tyrosine kinase 1 | F | GGATGGAGCAACCACAGCTTCTC |
| DDR1 | discoidin domain receptor tyrosine kinase 1 | R | GCATGTTGTTACAGTGGACCTGCAT |
| DiRas2 | DIRAS family, GTP-binding RAS-like 2 | F | CTGGTGTTGAGGTTTGTGAAAGG |
| DiRas2 | DIRAS family, GTP-binding RAS-like 2 | R | CCGTCGTGTCGGTGATCTG |
| DLL1 | delta-like 1 protein precursor; delta homolog | F | AGCTACACTTGCTCTTGCCGGC |
| DLL1 | delta-like 1 protein precursor; delta homolog | R | CGTGCAGCTCCCTCCGTTCTTA |
| DLL3 | delta-like 3 protein precursor; delta homolog | F | GGTGAATGCCGATGCCTAGAG |
| DLL3 | delta-like 3 protein precursor; delta homolog | R | GTGGTAGCAGAGGACGGGCC |
| DLL4 | delta-like 4 protein precursor; delta homolog | F | GCTGTGTAACGAATGCATCCCC |
| DLL4 | delta-like 4 protein precursor; delta homolog | R | GAGATCTTGGTCACAAAACAGGCCT |
| DPP6 | dipeptidyl-peptidase 6 | F | AAGCCCTACCACTATCCCAAGGC |
| DPP6 | dipeptidyl-peptidase 6 | R | TCAGGCGGCATCATCTCCA |
| DPYSL5 | dihydropyrimidinase-like 5 | F | GACGCTTATGAGAAGTGCCGA |
| DPYSL5 | dihydropyrimidinase-like 5 | R | CTCACCAGTGTCTCCATTTCTG |
| EBPL | emopamil binding protein-like | F | GGTCTCTGGCATTGTTCCTCA |
| EBPL | emopamil binding protein-like | R | AACAGGTAAAGCCAACAGTACAG |
| EDNRB | Endothelin receptor type B | F | CCATTTGGAGCTGAGATGTGTAAGC |
| EDNRB | Endothelin receptor type B | R | CCAAGAAGCAACAGCTCGATATCTG |
| EGFR | epidermal growth factor receptor | F | GCGTTCGGCACGGTGTATAA |
| EGFR | epidermal growth factor receptor | R | GGCTTTCGGAGATGTTGCTTC |
| EML4 | echinoderm microtubule associated protein like 4 | F | TCGCCTGTCAGCTCTTGAGT |
| EML4 | echinoderm microtubule associated protein like 4 | R | AGGCCACATGATCTTCAGAGATT |
| ERBB2 | v-erb-b2 erythroblastic leukemia viral oncogene homolog 2 | F | GACGAATTCTGCACAATGGCG |
| ERBB2 | v-erb-b2 erythroblastic leukemia viral oncogene homolog 2 | R | CGAAGCAGAGGTGGGTGTTATG |
| FABP7 | fatty acid binding protein 7, brain | F | CTCTCAGCACATTCAAGAACACG |
| FABP7 | fatty acid binding protein 7, brain | R | GCGAACAGCAACCACATCAC |
| FN1 | fibronectin 1 | F | GCAGTAACCACTATTCCTGCAC |
| FN1 | fibronectin 1 | R | TCCTGATACAACCACGGATGAG |
| FSTL1 | follistatin-like 1 | F | CAACCACTGTGAACTGCATCG |
| FSTL1 | follistatin-like 1 | R | CCTTTAGAGAACCAGCCATCTG |
| FTL | ferritin, light polypeptide | F | ACGAGCGTCTCCTGAAGATG |
| FTL | ferritin, light polypeptide | R | CCCAGGGCATGAAGATCCAAA |
| FXYD6 | FXYD domain containing ion transport regulator 6 | F | ACCCTGAGGATTGGGGGAC |
| FXYD6 | FXYD domain containing ion transport regulator 6 | R | CATTGGCGGTGATGAGGTT |
| GADD45A | Homo sapiens growth arrest and DNA-damage-inducible, alpha (GADD45A), mRNA | F | GAGAGCAGAAGACCGAAAGGA |
| GADD45A | Homo sapiens growth arrest and DNA-damage-inducible, alpha (GADD45A), mRNA | R | CACAACACCACGTTATCGGG |
| GDF8 | Growth differentiation factor 8 | F | GGAAACAGCTCCTAACATCAGC |
| GDF8 | Growth differentiation factor 8 | R | TGTCATCCCTCTGGACATCATAC |
| GLCCI1 | glucocorticoid induced transcript 1 | F | GCAGCTCACCTGAGAGACG |
| GLCCI1 | glucocorticoid induced transcript 1 | R | AGGAGGTTCGCCTTATTGTACTA |
| GLUD1 | glutamate dehydrogenase 1 | F | CTGGCTTGGCATACACAATG |
| GLUD1 | glutamate dehydrogenase 1 | R | GCTGTTCTCAGGTCCAATCC |
| GPM6B | glycoprotein M6B | F | AGACCTGCAAACTTGTGCCA |
| GPM6B | glycoprotein M6B | R | CCCACAGCCGCAGAATAAG |
| GRIA2 | glutamate receptor, ionotropic, AMPA 2 | F | TGTTGGAGTCCACGATGAACG |
| GRIA2 | glutamate receptor, ionotropic, AMPA 2 | R | GCAAGATTTACTGGGGTTCCTAA |
| GYG2 | glycogenin 2 | F | AGGTCACCTGAAGCAGTTTAGCC |
| GYG2 | glycogenin 2 | R | CCTGAAATATCCTACGAGCATCG |
| HDAC4 | Histone deacetylase 4 | F | CACGAGCACATCAAGCAACAA |
| HDAC4 | Histone deacetylase 4 | R | CAGTGGTTCAGATTCCGGTGG |
| HES1 | hairy and enhancer of split 1, | F | GAGAGGCGGCTAAGGTGTTTG |
| HES1 | hairy and enhancer of split 1, | R | CTGGTGTAGACGGGGATGAC |
| HES2 | hairy and enhancer of split 2 | F | TGGACAGGTTGAAGCTCTTGG |
| HES2 | hairy and enhancer of split 2 | R | CCTTTTATTCCCTGAGCCGAG |
| HES6 | hairy and enhancer of split 6 | F | CCTTGGTGACCAATGCCAG |
| HES6 | hairy and enhancer of split 6 | R | CCTGCAAGCCATCCATCAG |
| HEY2 | hairy/enhancer-of-split related with YRPW motif 2 | F | GGCGTCGGGATCGGATAAATA |
| HEY2 | hairy/enhancer-of-split related with YRPW motif 2 | R | AAGTAGCCTTTACCCCCTGTT |
| HLA-B | major histocompatibility complex, class I, B | F | TTCGTGAGGTTCGACAGCG |
| HLA-B | major histocompatibility complex, class I, B | R | TGTAGTAGCCGCGCAGGTTC |
| HMGN2 | High-mobility group nucleosomal binding domain 2 | F | TCCCTGACTTTGACACACATGG |
| HMGN2 | High-mobility group nucleosomal binding domain 2 | R | GATTGCCTGACACACTGGTAACC |
| IGF2 | Insulin-like growth factor 2 (somatomedin A) | F | CCTCCAGTTCGTCTGTGGG |
| IGF2 | Insulin-like growth factor 2 (somatomedin A) | R | CACGTCCCTCTCGGACTTG |
| IGFBP1 | insulin-like growth factor binding protein 1 | F | GAGAGCACGGAGATAA CTGAGG |
| IGFBP1 | insulin-like growth factor binding protein 1 | R | TTGGTGACATGGAGAGCCTTCG |
| IGFBP10 | insulin-like growth factor binding protein 10 | F | TGTGGAACTGGTATCTCCACACGAG |
| IGFBP10 | insulin-like growth factor binding protein 10 | F | ACCGCTCTGAAGGGGATCT |
| IGFBP10 | insulin-like growth factor binding protein 10 | R | ACTGATGTTTACAGTTGGGCTG |
| IGFBP10 | insulin-like growth factor binding protein 10 | R | TTTTCAGGCTGCTGTACACTGGCT |
| IGFBP2 | insulin-like growth factor binding protein 2 | F | GACAATGGCGATGACCACTCA |
| IGFBP2 | insulin-like growth factor binding protein 2 | R | GCTCCTTCATACCCGACTTGA |
| IGFBP3 | insulin-like growth factor binding protein 3 | F | AGAGCACAGATACCCAGAACT |
| IGFBP3 | insulin-like growth factor binding protein 4 | R | TGAGGAACTTCAGGTGATTCAGT |
| IGFBP4 | insulin-like growth factor binding protein 4 | F | CCCACGAGGACCTCTACATCA |
| IGFBP4 | insulin-like growth factor binding protein 4 | R | CGTCTTCCGGTCCACACAC |
| IGFBP5 | insulin-like growth factor binding protein 5 | F | CGGGGTTTGCCTCAACGAA |
| IGFBP5 | insulin-like growth factor binding protein 5 | R | TCTTGGGGGAGTAGGTCTCCT |
| IGFBP6 | insulin-like growth factor binding protein 6 | F | CTGACCATGACCCCCCACA |
| IGFBP6 | insulin-like growth factor binding protein 6 | R | CACTCCTGCCCCTCCCTCC |
| IGFBP7 | insulin-like growth factor binding protein 7 | F | GGTCCTTCCATAGTGACGCC |
| IGFBP7 | insulin-like growth factor binding protein 7 | R | TCTGAATGGCCAGGTTGTCC |
| IGFBP8 | insulin-like growth factor binding protein 8 | F | CAGCATGGACGTTCGTCTG |
| IGFBP8 | insulin-like growth factor binding protein 8 | R | CCAACCACGGTTTGGTCCTT |
| IGFBP9 | insulin-like growth factor binding protein 9 | F | CAATAGGAACCGTCAATGTGAGA |
| IGFBP9 | insulin-like growth factor binding protein 9 | R | CTTGGTGCGGAGACACTTTTT |
| IGFBPL1 | insulin-like growth factor binding protein-like 1 | F | GTCACGAAGTCCCCTGAGGG |
| IGFBPL1 | insulin-like growth factor binding protein-like 1 | R | CCGTGGCCTCATGGTCAGA |
| ITPKB | inositol 1,4,5-trisphosphate 3-kinase B | F | GACTTCAGGAACCGACTCCG |
| ITPKB | inositol 1,4,5-trisphosphate 3-kinase B | R | TTCTCCATCCTTACAAAAGCGG |
| JAG1 | jagged 1 | F | GTCCATGCAGAACGTGAACG |
| JAG1 | jagged 1 | R | GCGGGACTGATACTCCTTGA |
| JAG2 | jagged 2 | F | AGCTGGACGCCAATGAGTG |
| JAG2 | jagged 2 | R | GTCGTTGACGTTGATATGGCA |
| LAMB1 | Laminin, beta 1 | F | ACAAGCCCGAACCCTACTGTA |
| LAMB1 | Laminin, beta 1 | R | GACCACATTTTCAATGAGATGGC |
| LFNG | LFNG O-fucosylpeptide 3-beta-N-acetylglucosaminyltransferase | F | GACCACCAAAAAGTTCCACCG |
| LFNG | LFNG O-fucosylpeptide 3-beta-N-acetylglucosaminyltransferase | R | GGCCGAGCAGTTTGTGATGA |
| LGALS1 | lectin, galactoside-binding, soluble, 1 | F | CTCCTGACGCTAAGAGCTTCG |
| LGALS1 | lectin, galactoside-binding, soluble, 1 | R | CCAGGCTGGAAGGGAAAGAC |
| LGALS3 | lectin, galactoside-binding, soluble, 3 | F | TGCTGATAACAATTCTGGGCAC |
| LGALS3 | lectin, galactoside-binding, soluble, 3 | R | TGAAGCGTGGGTTAAAGTGGA |
| LIF | leukemia inhibitory factor (cholinergic differentiation factor) | F | GTACCGCATAGTCGTGTACCT |
| LIF | leukemia inhibitory factor (cholinergic differentiation factor) | R | CACAGCACGTTGCTAAGGAG |
| LOX | Lysyl oxidase | F | CAGGGTGCTGCTCAGATTTCC |
| LOX | Lysyl oxidase | R | GGTAATGTTGATGACAACTGTGC |
| MAL2 | Mal, T-cell differentiation protein 2 | F | TTGCCTCCTCCAATGTTCCTC |
| MAL2 | Mal, T-cell differentiation protein 2 | R | CAGTTAGCATCAATTTGAGCCAC |
| MBP | myelin basic protein | F | CTCTGGATCACCCATGGCTAG |
| MBP | myelin basic protein | R | GAAATTGCCGGTAGGCTGC |
| MCF2 | MCF.2 cell line derived transforming sequence | F | GGAGAGGCAAGATGAGGTTCA |
| MCF2 | MCF.2 cell line derived transforming sequence | R | CTCAGGGGTTAATTGCTTGTCA |
| MCM2 | minichromosome maintenance complex component 2 | F | ATGGCGGAATCATCGGAATCC |
| MCM2 | minichromosomemaintenance complex component 2 | R | GGTGAGGGCATCAGTACGC |
| MET | Met proto-oncogene (hepatocyte growth factor receptor) | F | TGGTGCAGAGGAGCAATGG |
| MET | Met proto-oncogene (hepatocyte growth factor receptor) | R | CATTCTGGATGGGTGTTTCCG |
| MGP | matrixGla protein | F | AGATGGAGAGCTAAAGTCCAAGA |
| MGP | matrixGla protein | R | GTAGCGTTCGCAAAGTCTGTA |
| MMP2 | matrixmetallopeptidase 2 | F | CCGTCGCCCATCATCAAGTT |
| MMP2 | matrixmetallopeptidase 2 | R | CTGTCTGGGGCAGTCCAAAG |
| MMP9 | matrixmetallopeptidase 9 | F | CATTTCGACGATGACGAGTTGT |
| MMP9 | matrixmetallopeptidase 9 | R | CGGGTGTAGAGTCTCTCGC |
| NCALD | neurocalcin delta | F | GACTTGCTGGAAAGCACAGAC |
| NCALD | neurocalcin delta | R | ATTTGGAAGCATCCCCATAAGG |
| NOTCH1 | Notch homolog 1 | F | GAGGCGTGGCAGACTATGC |
| NOTCH1 | Notch homolog 1 | R | CTTGTACTCCGTCAGCGTGA |
| NOTCH2 | Notch homolog 2 | F | CTGTGAGTGTCTGAAGGGTTATG |
| NOTCH2 | Notch homolog 2 | R | GGCACTGGAAACGATTGACTTT |
| NOTCH3 | Notch homolog 3 | R | CGTGGCTTCTTTCTACTGTGC |
| NOTCH3 | Notch homolog 3 | R | CGTTCACCGGATTTGTGTCAC |
| NOTCH4 | Homo sapiens Notch homolog 4 (Drosophila) (NOTCH4), mRNA | R | CTGGGTGTCAATGGAGAGGGA |
| NOTCH4 | Homo sapiens Notch homolog 4 (Drosophila) (NOTCH4), mRNA | R | GGGTGAGACGTGCCAGTTTC |
| NSEP1 | DNA-binding protein B(actuaslly WANT TO DESIGN FOR 'nuclease sensitive element binding protein 1) | F | AGCCACCTCAACGTCGGTA |
| NSEP1 | DNA-binding protein B(actuaslly WANT TO DESIGN FOR 'nuclease sensitive element binding protein 1) | R | TGGATCGGCTGCTTTTGTCTC |
| NTRK2 | Neurotrophic tyrosine kinase, receptor, type 2 | F | GATAAGCTGGACTCGGCACG |
| NTRK2 | Neurotrophic tyrosine kinase, receptor, type 2 | R | GGACGACATCCCTAGCAGCC |
| NUDT10 | Nudix (nucleoside diphosphate linked moiety X)-type motif 10 | F | CTCCTGGGCGTCTTCGAAC |
| NUDT10 | Nudix (nucleoside diphosphate linked moiety X)-type motif 10 | R | GCTAACCGAATCTTCCCAATCC |
| OCIL | C-type lectin superfamily 2, member D | F | GCTGCTTTAAGCGCAATAAGAG |
| OCIL | C-type lectin superfamily 2, member D | R | AACCTGAGCAAGATCAGCATC |
| OLFM1 | olfactomedin 1 | F | AGACCATGTGTTCACGGGATG |
| OLFM1 | olfactomedin 1 | R | AGGTGTTGCTTATGACTCTCCT |
| OLIG1 | oligodendrocyte transcription factor 1 | F | CGGACGCCAAGGAGGAGC |
| OLIG1 | oligodendrocyte transcription factor 1 | R | TATCTTGGAGAGCTTGCGGCC |
| OLIG2 | oligodendrocyte transcription factor 2 | F | GGACAAGCTAGGAGGCAGTG |
| OLIG2 | oligodendrocyte transcription factor 2 | R | ATGGCGATGTTGAGGTCGTG |
| PACSIN1 | Protein kinase C and casein kinase substrate in neurons 1 | F | CACCGTCTATGCAACGACCTG |
| PACSIN1 | Protein kinase C and casein kinase substrate in neurons 1 | R | GCCTCTGTCATTATGGCACCC |
| PBEF1 | Homo sapiens pre-B-cell colony enhancing factor 1 (PBEF1), transcript variant 1, Mrna | F | ATTGCCTTCGGTTCTGGTGG |
| PBEF1 | Homo sapiens pre-B-cell colony enhancing factor 1 (PBEF1), transcript variant 1, Mrna | R | CGGCCCTTTTTGGACCTTTTG |
| PCDHGA11 | protocadheringammasubfamily A, 11 isoform 1 precursor | F | TCAGACCTCTCGCTGTACTTG |
| PCDHGA11 | protocadheringammasubfamily A, 11 isoform 1 precursor | R | CCGTCCACGCCTACAAAATGT |
| PHLDA1 | pleckstrin homology-like domain, family A, member 1 | F | GGAGATCGACTTTCGGTGCC |
| PHLDA1 | pleckstrin homology-like domain, family A, member 1 | R | CTTCTGCCGCGTGGATTTG |
| PHLPP | PH domain and leucine rich repeat protein phosphatase | F | ACTGGGATTTGGGGAGCTG |
| PHLPP | PH domain and leucine rich repeat protein phosphatase | R | CGTCTTGTCCATCGGTTCACT |
| PLAT | Homo sapiens plasminogen activator, tissue (PLAT) | F | ACTGCCGGAATCCTGATGG |
| PLAT | Homo sapiens plasminogen activator, tissue (PLAT) | R | TGTGCTTGGCAAAGATGGC |
| PLEKHB1 | pleckstrin homology domain containing, family B (evectins) member 1 | F | AAAGATCGGCCCAGAGTGC |
| PLEKHB1 | pleckstrin homology domain containing, family B (evectins) member 1 | R | GGAGTTTGCCTCCAGCAGTG |
| PLEKHH2 | potassium channel tetramerisation domain containing 2 | F | CTCCGAAACCCTTATCACCATTC |
| PLEKHH2 | potassium channel tetramerisation domain containing 2 | R | TGAACAACGCAAATCCAGACTG |
| PTBP1 | polypyrimidine tract binding protein 1 | F | GAGCCCCGGTCCTATCTTAGAG |
| PTBP1 | polypyrimidine tract binding protein 1 | R | GCAGGCAAAACACAGAAATGAG |
| PTPN13 | Protein tyrosine phosphatase, non-receptor type 13 (APO-1/CD95 (Fas)-associated phosphatase) | F | ACTCCAGCAGTCAGGATCATC |
| PTPN13 | Protein tyrosine phosphatase, non-receptor type 13 (APO-1/CD95 (Fas)-associated phosphatase) | R | ATATCTCCAGGCTTAGGAGGTG |
| PTPRO | Protein tyrosine phosphatase, receptor type, O | F | TTCCATACAACTGGCGTAGGA |
| PTPRO | Protein tyrosine phosphatase, receptor type, O | R | CCAAAGACATGAGGGTAGCAG |
| PTPRZ1 | protein tyrosine phosphatase, receptor-type, Z polypeptide 1 | F | GCATGTTTCCAGGGTCCCTT |
| PTPRZ1 | protein tyrosine phosphatase, receptor-type, Z polypeptide 1 | R | GTCACCAGATGCTTGAGAGAC |
| PTTG1 | Pituitary tumor-transforming 1 | F | TGATCCTTGACGAGGAGAGAG |
| PTTG1 | Pituitary tumor-transforming 1 | R | GGTGGCAATTCAACATCCAGG |
| QKI | quaking homolog, KH domain RNA binding | F | CTCTGTCCTGTGGTCCCGTG |
| QKI | quaking homolog, KH domain RNA binding | R | AGATCTTGCGCTGAAAGTGCTG |
| RAB13 | Homo sapiens RAB13, member RAS oncogene family (RAB13) | F | ATAACTACTGCCTACTACCGTGG |
| RAB13 | Homo sapiens RAB13, member RAS oncogene family (RAB13) | R | CCATGTCACATTTGTTCCCCAG |
| RAB26 | RAB26 protein [Homo sapiens] | F | GTCTGCTGGTGCGATTCAAG |
| RAB26 | RAB26 protein [Homo sapiens] | R | GCATGGGTAACACTGCGGA |
| RAN | RAN, member RAS oncogene family | F | AGGACAGGAAAGTGAAGGCG |
| RAN | RAN, member RAS oncogene family | R | TGGCAACAAATTCCAAGTTAGG |
| RFNG | Homo sapiens radical fringe homolog (Drosophila) | F | TGTGTATGGTGATGGTGAGTCGG |
| RFNG | Homo sapiens radical fringe homolog (Drosophila) | R | CAGCATTGAAGCATGGCATGAG |
| RGS4 | Regulator of G-protein signalling 4 | F | CAAGCCGGAACATGCTAGAG |
| RGS4 | Regulator of G-protein signalling 4 | R | CGGGTTGACCAAATCAAGATAGA |
| RND2 | Rho family GTPase 2 | F | GCGAGCTTTGAGATCGACAAG |
| RND2 | Rho family GTPase 2 | R | CAGTGTTTCTGGTCGGCTAAT |
| RPL35A | ribosomal protein L35a | F | GGGTACAGCATCACTCGGA |
| RPL35A | ribosomal protein L35a | R | ACGCCCGAGATGAAACAG |
| RPLP1 | ribosomalprotein P1; 60S acidicribosomalprotein P1; acidicribosomalphosphoprotein P1 [Homo sapiens | F | ATTCTGCACGACGATGAGGTG |
| RPLP1 | ribosomalprotein P1; 60S acidicribosomalprotein P1; acidicribosomalphosphoprotein P1 | R | GATGAGGCTCCCAATGTTGAC |
| RPS19 | Homo sapiens ribosomalprotein S19 (RPS19) | F | AAAGAGCTTGCTCCCTACGAT |
| RPS19 | Homo sapiens ribosomalprotein S19 (RPS19) | R | CATGACGCCGTTTCTCTGAC |
| RPS3 | ribosomal protein S3 | F | TGCGAGGTTGTGGTGTCTG |
| RPS3 | ribosomal protein S3 | R | ACAGCAGTGTCAACGTAGTAGT |
| RTN3 | reticulon 3 | F | GGACGTAGACATTACTCTGTCCT |
| RTN3 | reticulon 3 | R | CACCAACATAGGTCATCAGCC |
| S100A4 | S100 calcium binding protein A4 (calcium protein, calvasculin, metastasin, murine placental homolog) | F | GATGAGCAACTTGGACAGCAA |
| S100A4 | S100 calcium binding protein A4 (calcium protein, calvasculin, metastasin, murine placental homolog) | R | CTGGGCTGCTTATCTGGGAAG |
| S100A6 | S100 calcium binding protein A6 (calcium protein, calvasculin, metastasin, murine placental homolog) | F | GGCTGATGGAAGACTTGGACC |
| S100A6 | S100 calcium binding protein A6 (calcium protein, calvasculin, metastasin, murine placental homolog) | R | TTTGACTCAGAGAGGACCCCC |
| SATB1 | Special AT-rich sequence binding protein 1 (binds to nuclear matrix/scaffold-associating DNA's) | F | ACAGGTGCAAAAATGCAGGGA |
| SATB1 | Special AT-rich sequence binding protein 1 (binds to nuclear matrix/scaffold-associating DNA's) | R | GCGTTTTCATAATGTTCCACCAC |
| SEMA4D | Homo sapiens sema domain, immunoglobulin domain (Ig), transmembrane domain (TM) and short cytoplasmic domain, (semaphorin) 4D SEMA4D), mRNA | F | TGTGACCACCTGAACTTAACATC |
| SEMA4D | Homo sapiens sema domain, immunoglobulin domain (Ig), transmembrane domain (TM) and short cytoplasmic domain, (semaphorin) 4D SEMA4D), mRNA | R | TCTCCATCAACCATGACGGAT |
| SHC3 | SHC (Src homology 2 domain containing) transforming protein 3 | F | ACCTCCAGTTTGCGGGAATG |
| SHC3 | SHC (Src homology 2 domain containing) transforming protein 3 | R | CGCATGTGGTGATTCGCTATG |
| SLC1A3 | solute carrier family 1 (glial high affinity glutamate transporter), member 3 | F | GAATGGCGGCGCTAGATAGTA |
| SLC1A3 | solute carrier family 1 (glial high affinity glutamate transporter), member 3 | R | GTGCATGTTTTCCTTTGTGCC |
| SMC5L1 | SMC5 protein | F | TGCCATTTGCCTTGGTTTAGC |
| SMC5L1 | SMC5 protein | R | CACGGGTGATTACAAGATTTCCA |
| SNCA | Synuclein, alpha (non A4 component of amyloid precursor) | F | ATGTAGGCTCCAAAACCAAGG |
| SNCA | Synuclein, alpha (non A4 component of amyloid precursor) | R | CCTCCAACATTTGTCACTTGCT |
| SNCB | Homo sapiens synuclein, beta (SNCB), transcript variant 1, mRNA | F | ATGAAGGGCCTGTCCATGG |
| SNCB | Homo sapiens synuclein, beta (SNCB), transcript variant 1, mRNA | R | TTCCGACGTAGAGGACGCC |
| SOD2 | Homo sapiens superoxide dismutase 2, mitochondrial (SOD2), nuclear gene encoding mitochondrial protein, transcript variant 1,2, 3 mRNA | F | AACCTCAGCCCTAACGGTG |
| SOD2 | Homo sapiens superoxide dismutase 2, mitochondrial (SOD2), nuclear gene encoding mitochondrial protein, transcript variant 1,2, 3 mRNA | R | AGCAGCAATTTGTAAGTGTCCC |
| SPARC | Homo sapiens secreted protein, acidic, cysteine-rich (osteonectin) (SPARC), mRNA | F | CGAGACCTGTGACCTGGACAATG |
| SPARC | Homo sapiens secreted protein, acidic, cysteine-rich (osteonectin) (SPARC), mRNA | R | TCCGGTACTGTGGAAGGAGTGG |
| SPARCL1 | SPARC-like 1; mast9; hevin [Homo sapiens]. | F | GCACCTGACAACACTGCAATC |
| SPARCL1 | SPARC-like 1; mast9; hevin [Homo sapiens]. | R | TTTCAGCCTTATGGTGGGAATC |
| SYT1 | Human synaptotagmin I mRNA, 3' UTR | F | GGTTGGCTGTTTCCCAGTAAAAC |
| SYT1 | Human synaptotagmin I mRNA, 3' UTR | R | TTTTAAGAAGTACGGACCATCGG |
| SYT5 | Synaptotagmin V | F | TGTTTCTGTCTCTACCGGAAGA |
| SYT5 | Synaptotagmin V | R | GCACCTTGTCTATGTAACTCTGG |
| TFAP2A | transcription factor AP-2 alpha | F | AGGTCAATCTCCCTACACGAG |
| TFAP2A | transcription factor AP-2 alpha | R | GGAGTAAGGATCTTGCGACTGG |
| TFAP2B | transcription factor AP-2 beta | F | GCCCCCTGAATGCCTCAAT |
| TFAP2B | transcription factor AP-2 beta | R | AGTAACGTGACATTTGCTGCTT |
| TFAP2C | transcription factor AP-2 gamma | F | CGGAGCGGTCTTGACACTC |
| TFAP2C | transcription factor AP-2 gamma | R | CAAGCGGTAAATCCAAAATCGG |
| TFAP2D | transcription factor AP-2 delta | F | TCCTTCCATTACGAGTTTCAGC |
| TFAP2D | transcription factor AP-2 delta | R | GCAGGTTAATAAAGTCGGTGGG |
| TFAP2E | transcription factor AP-2 epsilon | F | GGTGTTTGCGGGAACGGTTA |
| TFAP2E | transcription factor AP-2 epsilon | R | CTCTCCTTCCACTAGCGAAGT |
| TGFBR1 | transforming growth factor, beta receptor 1 | F | CCTAGGATGCTCACCTTCCAAG |
| TGFBR1 | transforming growth factor, beta receptor 1 | R | AGGTTCCAAGAAACAGCTGGAG |
| THBS1 | Thrombospondin 1 | F | CCTGACCGTCCAAGGAAAGC |
| THBS1 | Thrombospondin 1 | R | CCTTTGCGATGCGGAGTCT |
| THBS2 | thrombospondin 2 | F | ACAAAGACACGACCTTCGACC |
| THBS2 | thrombospondin 2 | R | GACTTGCCGTCCTGCTTGA |
| THBS4 | thrombospondin 4 | F | TGCTGCCAGTCCTGACAGA |
| THBS4 | thrombospondin 4 | R | GTTTAAGCGTCCCATCACAGTA |
| TIAM1 | Homo sapiens T-cell lymphoma invasion and metastasis 1 (TIAM1), | F | GATCCACAGGAACTCCGAAGT |
| TIAM1 | Homo sapiens T-cell lymphoma invasion and metastasis 1 (TIAM1), | R | GCTCCCGAAGTCTTCTAGGGT |
| TIMP1 | Tissue inhibitor of metalloproteinase 1 (erythroid potentiating activity, collagenase inhibitor) | F | CACCAGAGAACCCACCATGG |
| TIMP1 | Tissue inhibitor of metalloproteinase 1 (erythroid potentiating activity, collagenase inhibitor) | R | GCTGGTATAAGGTGGTCTGGTTG |
| TOP2A | Topoisomerase (DNA) II alpha 170kDa | F | CTCGCTTGTCATTCCGTTTGA |
| TOP2A | Topoisomerase (DNA) II alpha 170kDa | R | CAACGAAAGTTACTTGGGCTTCC |
| TRIM9 | tripartite motif containing 9 | F | TGGTTCATGCACAACAACTCG |
| TRIM9 | tripartite motif containing 9 | R | CCCTCCACGTTATCAAATGCT |
| UEST 275430 | Homo sapiens cDNA FLJ41396 fis, clone BRCOC2019255 | F | ATGAATTGTCGGTTGTCCTGC |
| UEST 275430 | Homo sapiens cDNA FLJ41396 fis, clone BRCOC2019255 | R | AATTTTCCCCCACCTGTTCTG |
| UEST 39152 | Homo sapiens cDNA clone | F | GACATTTACGCCACAACCCAC |
| UEST 39152 | Homo sapiens cDNA clone | R | AGACCCTCAGATTCCCCACC |
| VEGFA | vascular endothelial growth factor A | F | CAACATCACCATGCAGATTATGC |
| VEGFA | vascular endothelial growth factor A | R | TCGGCTTGTCACATTTTTCTTGT |
| VIM | vimentin | F | CAGGAACAGCATGTCCAAATCG |
| VIM | vimentin | R | TGTACCATTCTTCTGCCTCCTGC |
| VPS13D | vacuolar protein sorting 13 homolog D (S. cerevisiae) | F | CGATGCCTCTTGCTGGAATG |
| VPS13D | vacuolar protein sorting 13 homolog D (S. cerevisiae) | R | TCTCTCGGATGTCTCTCTGCAG |
| VSNL1 | Visinin-like 1 | F | ATGGGGAAGCAGAATAGCAAAC |
| VSNL1 | Visinin-like 1 | R | CTCCCACTTGGACAGTCCTTG |
| YES1 | Yamaguchi sarcoma viral oncogenehomolog 1 | F | TTTTTGAGACGGAGTCTCGCTC |
| YES1 | Yamaguchi sarcoma viral oncogenehomolog 1 | R | CACGGTGAAACTGCGTCTCTAC |
| ZNF224 | Zinc finger protein 224/Homo sapiens zinc finger protein 2 mRNA, complete cds | F | TTCAGAAGCAGGAACACATCAAG |
| ZNF224 | Zinc finger protein 224/Homo sapiens zinc finger protein 2 mRNA, complete cds | R | GGATGGTTTATATCCAATTGCCC |
| ZNF35 | zinc finger protein 35 (clone HF.10); Zinc finger protein-35 (HF.10) | F | GCCTGTAACGACTGTGGCAA |
| ZNF35 | zinc finger protein 35 (clone HF.10); Zinc finger protein-35 (HF.10) | R | ACAATAAGGCAAGAGAGCTGACT |

*F/R- Forward/Reverse primer
