# Supplementary material for: A Fourteen Gene GBM Prognostic Signature Identifies Association of Immune Response Pathway and Mesenchymal Subtype with High Risk Group
Source: PLoS One. 2013 Apr 30;8(4):e62042. doi: 10.1371/journal.pone.0062042 (PMC3639942; doi:10.1371/journal.pone.0062042)
Supplement: Table S2 — List of genes selected for expression analysis by RT-qPCR. (DOCX) [file pone.0062042.s004.docx]

**Supplementary table S2:** List of genes selected for expression analysis by RT-qPCR

| **S.No.** | **Symbol** | **UGRepAcc** | **GENE FULL NAME** |
| --- | --- | --- | --- |
| 1 | DiRas2 | NM_017594 | DIRAS family, GTP-binding RAS-like 2 |
| 2 | MMP2 | NM_001127891 | matrixmetallopeptidase 2 |
| 3 | PTBP1 | NM_002819.3 | polypyrimidine tract binding protein 1 |
| 4 | TGFBR1 | NM_001130916 | transforming growth factor, beta receptor 1 |
| 5 | UEST | NM_002819.3 | Homo sapiens cDNA clone nav32d02 5-, mRNAsequence |
| 6 | YES1 | NM_005433 | Yamaguchi sarcoma viral oncogene homolog1 |
| 7 | DCN | NM_001920 | decorin |
| 8 | LGALS3 | NM_001177388 | lectin, galactoside-binding, soluble, 3 |
| 9 | VEGFA | NM_001025366 | vascular endothelial growth factor A |
| 10 | FN1 | NM_002026 | fibronectin 1 |
| 11 | MMP9 | NM_004994 | matrixmetallopeptidase 9 |
| 12 | MGP | NM_000900 | matrixGla protein |
| 13 | THBS2 | NM_003247 | thrombospondin 2 |
| 14 | TFAP2C | NM_003222 | transcription factor AP-2 gamma |
| 15 | TFAP2D | NM_172238 | transcription factor AP-2 delta |
| 16 | TFAP2E | NM_178548 | transcription factor AP-2 epsilon |
| 17 | TFAP2B | NM_003221 | transcription factor AP-2 beta |
| 18 | ADD3 | NM_001121 | adducin 3 |
| 19 | AEBP1 | NM_001129 | AE binding protein 1 |
| 20 | AGT | NM_000029 | angiotensinogen |
| 21 | AQUA | NM_198098.1 | Homo sapiens aquaporin 1 (channel-forming integral protein, 28kDa) |
| 22 | ARC | BAA19667 | activity-regulated cytoskeleton-associated protein |
| 23 | ARHC | NM_175744.3 | ras homolog gene family, member C |
| 24 | ASCL1 | NM_004316 | achaete-scute complex homolog 1 |
| 25 | B2M | NM_004048 | beta-2-microglobulin |
| 26 | BCAN | NM_021948 | brevican |
| 27 | BHC80 | NM_001101802 | PHD finger protein 21A |
| 28 | BMP2 | NM_001200 | bone morphogenetic protein 2 |
| 29 | C1QB | NM_000491 | complement component 1, q subcomponent, B chain |
| 30 | C5ORF18 | NM_005669 | polyposis locus protein 1; deleted in polyposis 1; polyposis coli region hypothetical protein DP1 [Homo sapiens]. |
| 31 | C6ORF66 | NM_014165 | NADH dehydrogenase (ubiquinone) 1 alpha subcomplex, assembly factor 4 |
| 32 | CACNG4 | NM_014405 | calciumchannel, voltage-dependent, gamma subunit4 |
| 33 | CALCRL | NM_005795 | calcitonin receptor-like |
| 34 | CBX3 | NM_007276 | chromobox homolog 3 |
| 35 | CCL2 | NM_002982 | chemokine (C-C motif) ligand 2 |
| 36 | CD99L2 | NM_001184808 | CD99 molecule-like 2 |
| 37 | CDC2 | NM_001786 | cell division cycle 2, G1 to S and G2 to M |
| 38 | CDKN3 | NM_001130851 | cyclin-dependent kinase inhibitor 3 |
| 39 | CENPF | NM_016343 | centromere protein F |
| 40 | CHI3L1 | NM_001276 | chitinase 3-like 1 |
| 41 | CITED1 | NM_001144885 | Cbp/p300-interacting transactivator, with Glu/Asp-rich carboxy-terminal domain, 1 |
| 42 | CLU | NM_203339 | clusterin |
| 43 | CNR1 | NM_001160226 | cannabinoid receptor 1 |
| 44 | COL4A2 | NM_001846.1 | collagen, type IV |
| 45 | COL6A1 | NM_001848 | collagen, type VI, alpha 1 |
| 46 | COL6A3 | NM_004369 | collagen, type VI, alpha 3 |
| 47 | CPE | NM_001873 | carboxypeptidase E |
| 48 | CRB1 | NM_001193640 | crumbs homolog 1 |
| 49 | CRTAC1 | NM_001206528 | cartilage acidic protein 1 |
| 50 | CRYAB | NM_001885 | crystallin, alpha B |
| 51 | CSDC2 | NM_014460 | cold shock domain containing C2, RNA binding |
| 52 | CX43(MAG17) | NM_000165.2 | connexin 43 |
| 53 | DBI | NM_001079862 | diazepam binding inhibitor |
| 54 | DCX | NM_000555 | doublecortin |
| 55 | DDR1 | NM_001202521 | discoidin domain receptor tyrosine kinase 1 |
| 56 | DLL1 | NM_005618 | delta-like 1 protein precursor; delta homolog [Homo sapiens]. |
| 57 | DLL3 | NM_016941 | delta-like 3 protein precursor; delta homolog [Homo sapiens]. |
| 58 | DLL4 | NM_019074 | delta-like 4 protein precursor; delta homolog [Homo sapiens]. |
| 59 | DPP6 | NM_001039350 | dipeptidyl-peptidase 6 |
| 60 | DPYSL5 | NM_020134 | dihydropyrimidinase-like 5 |
| 61 | EBPL | NM_032565 | emopamil binding protein-like |
| 62 | EDNRB | NM_000115 | Endothelin receptor type B |
| 63 | EGFR | NM_005228 | epidermal growth factor receptor |
| 64 | EML4 | NM_001145076 | echinoderm microtubule associated protein like 4 |
| 65 | ERBB2 | NM_001005862 | v-erb-b2 erythroblastic leukemia viral oncogene homolog 2 |
| 66 | FABP7 | NM_001446 | fatty acid binding protein 7, brain  [Homo sapiens] |
| 67 | FLJ10619 | AI378412 | Homo sapiens vacuolar protein sorting 13D (yeast) (VPS13D) |
| 68 | FSTL1 | NM_007085 | follistatin-like 1 |
| 69 | FTL | NM_000146 | ferritin, light polypeptide |
| 70 | FXYD6 | NM_022003 | FXYD domain containing ion transport regulator 6 |
| 71 | GADD45A | NM_001199741 | Homo sapiens growth arrest and DNA-damage-inducible, alpha (GADD45A), mRNA |
| 72 | GDF8 | NM_005259 | Growth differentiation factor 8 |
| 73 | GLCCI1 | BC050291 | glucocorticoid induced transcript 1 |
| 74 | GLUD1 | NM_005271 | glutamate dehydrogenase 1 |
| 75 | GPM6B | NM_001001994 | glycoprotein M6B |
| 76 | GRIA2 | NM_000826 | glutamate receptor, ionotropic, AMPA 2 |
| 77 | GYG2 | NM_003918.1 | glycogenin 1 |
| 78 | HDAC4 | NM_006037 | Histone deacetylase 4 |
| 79 | HES1 | NM_005524 | hairy and enhancer of split 1, |
| 80 | HES2 | NM_019089 | hairy and enhancer of split 2 |
| 81 | HES6 | NM_001142853 | hairy and enhancer of split 6 |
| 82 | HEY2 | NM_012259 | hairy/enhancer-of-split related with YRPW motif 2 |
| 83 | HLA-B | NM_005514 | major histocompatibility complex, class I, B |
| 84 | HMGN | NM_005517.2 | High-mobility group nucleosomal binding domain 2 |
| 85 | IGF2 | NM_000612 | Insulin-like growth factor 2 (somatomedin A) |
| 86 | IGFBP1 | NM_000596 | insulin-like growth factor binding protein 1 [Homo sapiens]. |
| 87 | IGFBP10 | NM_001554 | insulin-like growth factor binding protein 10 [Homo sapiens]. |
| 88 | IGFBP2 | NM_000597 | insulin-like growth factor binding protein 2 [Homo sapiens]. |
| 89 | IGFBP3 | NM_000598 | insulin-like growth factor binding protein 3 [Homo sapiens]. |
| 90 | IGFBP4 | NM_001552 | insulin-like growth factor binding protein 4 [Homo sapiens]. |
| 91 | IGFBP5 | NM_000599 | insulin-like growth factor binding protein 5 [Homo sapiens]. |
| 92 | IGFBP6 | NM_002178 | insulin-like growth factor binding protein 6 [Homo sapiens]. |
| 93 | IGFBP7 | NM_001553 | insulin-like growth factor binding protein 7 [Homo sapiens]. |
| 94 | IGFBP8 | NM_001901 | insulin-like growth factor binding protein 8 [Homo sapiens]. |
| 95 | IGFBP9 | NM_002514 | insulin-like growth factor binding protein 9 [Homo sapiens]. |
| 96 | IGFBPL1 | NM_001007563 | insulin-like growth factor binding protein-like 1 |
| 97 | IL1RL1 | NM_003856.2 | interleukin 1 receptor-like 1 |
| 98 | ITPKB | NM_002221 | inositol 1,4,5-trisphosphate 3-kinase B |
| 99 | JAG1 | NM_000214.1 | jagged 1 |
| 100 | JAG2 | NM_002226 | jagged 2 |
| 101 | KIAA2028 | NM_172069.1 | potassium channel tetramerisation domain containing 2 |
| 102 | KIAA0773 | BC045611 | KIAA0773 gene product [Homo sapiens]. |
| 103 | KIAA1102 | NM_005109 | KIAA1102 protein [Homo sapiens] |
| 104 | LAMB1 | NM_002291 | Laminin, beta 1 |
| 105 | LFNG | NM_001040167 | LFNG O-fucosylpeptide 3-beta-N-acetylglucosaminyltransferase |
| 106 | LGALS1 | NM_002305 | lectin, galactoside-binding, soluble, 1 (galectin 1) |
| 107 | LIF | NM_002309 | leukemia inhibitory factor (cholinergic differentiation factor) |
| 108 | LOX | NM_001178102 | Lysyl oxidase |
| 109 | MAL2 | NM_052886 | Mal, T-celldifferentiationprotein 2 |
| 110 | MBP | NM_001025081 | myelin basic protein |
| 111 | MCF2 | NM_001099855 | MCF.2 cell line derived transforming sequence |
| 112 | MCM2 | NM_004526 | minichromosome maintenance complex component 2 |
| 113 | MET | NM_000245 | Met proto-oncogene (hepatocyte growth factor receptor) |
| 114 | MFNG | NM_001166343 | MFNG O-fucosylpeptide 3-beta-N-acetylglucosaminyltransferase |
| 115 | NCALD | NM_032041 | neurocalcin delta  [Homo sapiens] |
| 116 | NOTCH2 | NM_001200001 | Notch homolog 2 [Homo sapiens]. |
| 117 | NOTCH3 | NM_000435 | Notch homolog 3 [Homo sapiens]. |
| 118 | NOTCH4 | NM_004557 | Homo sapiens Notch homolog 4 (Drosophila) (NOTCH4), mRNA |
| 119 | NSEP1 | NM_004559 | DNA-binding protein B(actuaslly WANT TO DESIGN FOR 'nuclease sensitive element binding protein 1) |
| 120 | NTRK2 | NM_001007097 | Neurotrophic tyrosine kinase, receptor, type 2 |
| 121 | NUDT10 | NM_153183 | Nudix (nucleoside diphosphate linked moiety X)-type motif 10 |
| 122 | OCIL | NM_013269 | C-type lectin superfamily 2, member D |
| 123 | OLFM1 | NM_006334 | olfactomedin related ER localized protein isoform 1; neuroblastoma protein; olfactomedin related ER localized protein; pancortin 1 |
| 124 | OLIG1 | NM_138983 | oligodendrocyte transcription factor 1 |
| 125 | OLIG2 | NM_005806 | oligodendrocyte transcription factor 2 |
| 126 | PACSIN1 | NM_001199583 | Protein kinase C and casein kinase substrate in neurons 1 |
| 127 | PBEF1 | NM_005746.1 | Homo sapiens pre-B-cell colony enhancing factor 1 (PBEF1), transcript variant 1, Mrna |
| 128 | PCDHGA11 | NM_018914 | protocadheringammasubfamily A, 11 isoform 1 precursor |
| 129 | PHLDA1 | NM_007350 | pleckstrin homology-like domain, family A, member 1 |
| 130 | PHLPP | AB011178 | PH domain and leucine rich repeat protein phosphatase |
| 131 | PLAT | NM_000930 | Homo sapiens plasminogen activator, tissue (PLAT) |
| 132 | PLEKHB1 | NM_001130033 | pleckstrin homology domain containing, family B (evectins) member 1 |
| 133 | PTPN13 | NM_006264 | Protein tyrosine phosphatase, non-receptor type 13 (APO-1/CD95 (Fas)-associated phosphatase) |
| 134 | PTPRO | NM_002848 | Protein tyrosine phosphatase, receptor type, O |
| 135 | PTPRZ1 | NM_001206838 | protein tyrosine phosphatase, receptor-type, Z polypeptide 1 |
| 136 | PTTG1 | NM_004219 | Pituitary tumor-transforming 1 |
| 137 | QKI | NM_006775 | quaking homolog, KH domain RNA binding |
| 138 | RAB13 | NM_002870 | Homo sapiens RAB13, member RAS oncogene family (RAB13) |
| 139 | RAB26 | NM_014353 | RAB26 protein [Homo sapiens] |
| 140 | RAN | NM_006325 | RAN, member RAS oncogene family |
| 141 | RFNG | NM_002917 | Homo sapiens radical fringe homolog (Drosophila) |
| 142 | RGS4 | NM_001102445 | Regulator of G-protein signalling 4 |
| 143 | RND2 | NM_005440 | Rho family GTPase 2 |
| 144 | RPLP1 | NM_001003 | ribosomalprotein P1; 60S acidicribosomalprotein P1; acidicribosomalphosphoprotein P1 [Homo sapiens |
| 145 | RPS19 | NM_001022 | Homo sapiens ribosomalprotein S19 (RPS19) |
| 146 | RPS3 | NM_001005 | ribosomalprotein S3; 40S ribosomalprotein S3 [Homo sapiens]. |
| 147 | RTN3 | NM_006054 | reticulon 3 [Homo sapiens] |
| 148 | S100A4 | NM_002961 | S100 calcium binding protein A4 (calcium protein, calvasculin, metastasin, murine placental homolog) |
| 149 | S100A6 | NM_014624 | S100 calcium binding protein A6 (calcium protein, calvasculin, metastasin, murine placental homolog) |
| 150 | SATB1 | NM_001131010 | Special AT-rich sequence binding protein 1 (binds to nuclear matrix/scaffold-associating DNA's) |
| 151 | SEMA4D | NM_001142287 | Homo sapiens sema domain, immunoglobulin domain (Ig), transmembrane domain (TM) and short cytoplasmic domain, (semaphorin) 4D SEMA4D), mRNA |
| 152 | SHC3 | NM_016848 | SHC (Src homology 2 domain containing) transforming protein 3 |
| 153 | SLC1A3 | NM_001166695 | solute carrier family 1 (glial high affinity glutamate transporter), member 3 [Homo sapiens] |
| 154 | SMC5L1 | NM_015110 | SMC5 protein (Homo sapiens) |
| 155 | SNCA | NM_000345 | Synuclein, alpha (non A4 component of amyloid precursor) |
| 156 | SNCB | NM_001001502 | Homo sapiens synuclein, beta (SNCB), transcript variant 1, mRNA |
| 157 | SOD2 | NM_000636 | Homo sapiens superoxide dismutase 2, mitochondrial (SOD2), nuclear gene encoding mitochondrial protein, transcript variant 1,2, 3 mRNA |
| 158 | SPARC | NM_003118 | Homo sapiens secreted protein, acidic, cysteine-rich (osteonectin) (SPARC), mRNA |
| 159 | SPARCL1 | NM_001128310 | SPARC-like 1; mast9; hevin [Homo sapiens]. |
| 160 | SYT1 | NM_001135805 | Human synaptotagmin I mRNA, 3' UTR |
| 161 | SYT5 | NM_003180 | Synaptotagmin V |
| 162 | TFAP2a | NM_001032280 | transcription factor AP-2 alpha |
| 163 | THBS1 | NM_003246 | Thrombospondin 1 |
| 164 | THBS4 | NM_003248 | thrombospondin 4 [Homo sapiens] |
| 165 | TIAM1 | NM_003253 | Homo sapiens T-cell lymphoma invasion and metastasis 1 (TIAM1), |
| 166 | TIMP1 | NM_003254 | Tissue inhibitor of metalloproteinase 1 (erythroid potentiating activity, collagenase inhibitor) |
| 167 | TOP2A | NM_001067 | Topoisomerase (DNA) II alpha 170kDa |
| 168 | TRIM2 | NM_001130067 | tripartite motif-containing 2 |
| 169 | UEST_39152 |  | Homo sapiens cDNA clone |
| 170 | UEST_27543 | AK123390.1 | Homo sapiens cDNA FLJ41396 fis, clone BRCOC2019255 |
| 171 | UEST_f_218 | T50536.1 | yb30c07.r1 Stratagene fetal spleen (#937205) Homo sapiens cDNA |
| 172 | VIM | NM_003380 | vimentin |
| 173 | VSNL1 | NM_003385 | Visinin-like 1 |
| 174 | ZNF224 | NM_013398 | Zinc finger protein 224/Homo sapiens zinc finger protein 2 mRNA, complete cds |
| 175 | ZNF35 | NM_003420 | zinc finger protein 35 (clone HF.10); Zinc finger protein-35 (HF.10) [Homo sapiens]. |
| 176 | AGPAT* | NM_006411.2 | acylglycerol-3-phosphate O-acyltransferase |
| 177 | ATP5G1* | NM_005175.2 | ATP synthase, H+ transporting, mitochondrial Fo complex, subunit C1 (subunit 9) |
| 178 | GARS* | NM_002047.1 | glycyl-tRNAsynthetase (GARS) |
| 179 | RPL35* | NM_007209 | ribosomal protein L35 |
| 180 | 18S rRNA* | X03205 | 18S ribosomal RNA |
|  | | |  |

* Used as internal controls
