# Supplementary material for: A Fourteen Gene GBM Prognostic Signature Identifies Association of Immune Response Pathway and Mesenchymal Subtype with High Risk Group
Source: PLoS One. 2013 Apr 30;8(4):e62042. doi: 10.1371/journal.pone.0062042 (PMC3639942; doi:10.1371/journal.pone.0062042)
Supplement: Table S3 — Case bias analysis in the present data set. (DOCX) [file pone.0062042.s005.docx]

**Supplementary table S3:** Case bias analysis in the present data set.

| **Factor** | **Cases not included**  **for analysis (n=31)** | **Cases included for**  **analysis (n=123)** | **P Value** |
| --- | --- | --- | --- |
| Age (years) | 46**^.^**48 ± 13**^.^**01 | 47**^.^**23 ± 2**^.^**05 | 0**^.^**696 |
| Duration of symptom (days) | 88**^.^**73 ± 147**^.^**31 | 74**^.^**54 ± 68**^.^**8 | 0**^.^**545 |
| Median survival duration (months) | 16**^.^**3 ± 10**^.^**59 | 17**^.^**32 ± 11**^.^**56 | 0**^.^**899 |
| Maximum follow up (months) | 41(12**^.^**1) | 32(2**^.^**6) | 0**^.^**724 |
