# Supplementary material for: A Fourteen Gene GBM Prognostic Signature Identifies Association of Immune Response Pathway and Mesenchymal Subtype with High Risk Group
Source: PLoS One. 2013 Apr 30;8(4):e62042. doi: 10.1371/journal.pone.0062042 (PMC3639942; doi:10.1371/journal.pone.0062042)
Supplement: Table S4 — Genes selected from full data during cross validation. (DOCX) [file pone.0062042.s006.docx]

**Supplementary table S4:** Genes selected from full data during cross validation

| **Gene names** | **Importance-score** | **Raw-score** | **Median rank in CV** | **Proportion selected in CV** |
| --- | --- | --- | --- | --- |
| AGT | 426.772 | 1.083 | 2 | 0.77 |
| EGFR | 414.93 | 0.863 | 93 | 0.34 |
| CHI3L1 | 371.632 | 1.063 | 4 | 0.69 |
| SOD2 | 303.236 | 1.064 | 6 | 0.74 |
| CCL2 | 253.359 | 1.109 | 7 | 0.91 |
| IGFBPL1 | -224.725 | -1.42 | 6 | 1 |
| MBP | 191.301 | 1.584 | 7 | 1 |
| CPE | 189.754 | 0.91 | 93 | 0.43 |
| OLFM1 | 177.486 | 1.037 | 12 | 0.66 |
| MCF2 | -127.923 | -0.873 | 94 | 0.4 |
| PACSIN1 | 108.425 | 1.014 | 11 | 0.66 |
| CALCRL | 68.191 | -0.911 | 93 | 0.4 |
| SNCA | 67.731 | 1.184 | 11 | 0.86 |
| TOP2A | 60.353 | -1.041 | 16 | 0.74 |
