# Supplementary material for: A Fourteen Gene GBM Prognostic Signature Identifies Association of Immune Response Pathway and Mesenchymal Subtype with High Risk Group
Source: PLoS One. 2013 Apr 30;8(4):e62042. doi: 10.1371/journal.pone.0062042 (PMC3639942; doi:10.1371/journal.pone.0062042)
Supplement: Table S5 — Comparison of gene expression in present data set and TCGA data set. (DOCX) [file pone.0062042.s007.docx]

**Supplementary table S5:** Comparison of gene expression in present data set and TCGA data set

| **Sl**  **No** | **Gene**  **name** | **Present cohort (PCR array)** | | | | **TCGA** | | | |
| --- | --- | --- | --- | --- | --- | --- | --- | --- | --- |
|  |  | **Normal (Mean±SD)** | **GBM (Mean±SD)** | **P-value^*^** | **Regulation** | **Normal(Mean±SD)** | **GBM (Mean±SD)** | **P-value^*^** | **Regulation** |
| 1 | AGT | -0**^.^**085±0**^.^**887 | -1**^.^**683±3**^.^**886 | 0**^.^**1415 | unregulated | 7**^.^**451e-10±0**^.^**202 | -0**^.^**018±1**^.^**350 | 0**^.^**6110 | unregulated |
| 2 | EGFR | 0**^.^**001±0**^.^**733 | 2**^.^**317±3**^.^**172 | < 0**^.^**0001 | upregulated | -1**^.^**480e-8±0**^.^**253 | 2**^.^**378±2**^.^**171 | < 0**^.^**0001 | upregulated |
| 3 | CHI3L1 | -0**^.^**001±1**^.^**097 | 5**^.^**205±3**^.^**051 | < 0**^.^**0001 | upregulated | -1**^.^**863e-8±0**^.^**5292 | 1**^.^**915±1**^.^**286 | < 0**^.^**0001 | upregulated |
| 4 | SOD2 | 0**^.^**001000±0**^.^**6761 | 1**^.^**795±2**^.^**104 | < 0**^.^**0001 | upregulated | 8**^.^**941e-9±0**^.^**414 | 2**^.^**108±1**^.^**264 | < 0**^.^**0001 | upregulated |
| 5 | CCL2 | -0**^.^**001±1**^.^**845 | 0**^.^**562±2**^.^**112 | 0**^.^**2218 | unregulated | 1**^.^**788e-8±1**^.^**976 | 1**^.^**283±1**^.^**560 | 0**^.^**0627 | unregulated |
| 6 | IGFBPL1 | -0**^.^**0005000±1**^.^**983 | 0**^.^**5948±3**^.^**188 | 0**^.^**3785 | unregulated | -2**^.^**049e-9±0**^.^**147 | 0**^.^**010±0**^.^**510 | 0**^.^**3710 | unregulated |
| 7 | MBP | -3**^.^**912e-9±1**^.^**402 | -5**^.^**873±3**^.^**048 | < 0**^.^**0001 | downregulated | -1**^.^**397e-9±0**^.^**215 | -2**^.^**131±1**^.^**181 | < 0**^.^**0001 | downregulated |
| 8 | CPE | 2**^.^**980e-9±0**^.^**817 | -1**^.^**657±1**^.^**978 | < 0**^.^**0001 | downregulated | 2**^.^**980e-9±0**^.^**225 | -1**^.^**535±1**^.^**061 | < 0**^.^**0001 | downregulated |
| 9 | OLFM1 | -0**^.^**001±0**^.^**650 | -4**^.^**069±1**^.^**997 | < 0**^.^**0001 | downregulated | -4**^.^**657e-10±0**^.^**148 | -2**^.^**299±0**^.^**9728 | < 0**^.^**0001 | downregulated |
| 10 | MCF | 0**^.^**001±0**^.^**872 | -2**^.^**862±2**^.^**513 | < 0**^.^**0001 | downregulated | -8**^.^**382e-9±0**^.^**672 | -3**^.^**726±1**^.^**323 | < 0**^.^**0001 | downregulated |
| 11 | PACSIN1 | 0**^.^**001±0**^.^**609 | -6**^.^**332±2**^.^**466 | < 0**^.^**0001 | downregulated | 0**^.^**000±0**^.^**3011 | -4**^.^**553±1**^.^**544 | < 0**^.^**0001 | downregulated |
| 12 | CALCRL | 0**^.^**163±0**^.^**733 | 1**^.^**450±2**^.^**465 | < 0**^.^**0001 | upregulated | -7**^.^**451e-10±0**^.^**286 | 1**^.^**948±1**^.^**210 | < 0**^.^**0001 | upregulated |
| 13 | SNCA | 0**^.^**002±0**^.^**639 | -4**^.^**027±1**^.^**637 | < 0**^.^**0001 | downregulated | 7**^.^**451e-10±0**^.^**161 | -4**^.^**107±1**^.^**288 | < 0**^.^**0001 | downregulated |
| 14 | TOP2A | 0**^.^**001±1**^.^**287 | 8**^.^**277±2**^.^**116 | < 0**^.^**0001 | upregulated | -3**^.^**073e-9±0**^.^**205 | 2**^.^**866±1**^.^**195 | < 0**^.^**0001 | upregulated |

* P-value from Mann-Whitney test that shows the difference between normals and GBMs; SD- standard deviation
