# Supplementary material for: A Fourteen Gene GBM Prognostic Signature Identifies Association of Immune Response Pathway and Mesenchymal Subtype with High Risk Group
Source: PLoS One. 2013 Apr 30;8(4):e62042. doi: 10.1371/journal.pone.0062042 (PMC3639942; doi:10.1371/journal.pone.0062042)
Supplement: Table S6 — Univariate and multivariate analysis of WG score with other signatures. (DOCX) [file pone.0062042.s008.docx]

**Supplementary table S6:** Univariate and multivariate analysis of WG score with other signatures

| **Signature** | **HR** | **B (co-efficient)** | **P value** |
| --- | --- | --- | --- |
| **Univariate analysis** | | | |
| 4-gene signature | 1.1599 | 0.14833 | 0.0595 |
| 9-gene signature | 1.41785 | 0.34914 | <0.0001 |
| WG score | 1.9228 | 0.6538 | 0.00197 |
| **Multivariate analysis** | | | |
| 4-gene signature | 1.1999 | 0.1822 | 0.02174 |
| WG score | 1.8473 | 0.6137 | 0.00438 |
|  | | | |
| 9-gene signature | 1.35635 | 0.3048 | 0.000776 |
| WG score | 1.17746 | 0.16336 | 0.529216 |
