# Supplementary material for: A Fourteen Gene GBM Prognostic Signature Identifies Association of Immune Response Pathway and Mesenchymal Subtype with High Risk Group
Source: PLoS One. 2013 Apr 30;8(4):e62042. doi: 10.1371/journal.pone.0062042 (PMC3639942; doi:10.1371/journal.pone.0062042)
Supplement: Table S7 — Survival based on risk stratification by SWG score. (DOCX) [file pone.0062042.s009.docx]

**Supplementary table S7:** Survival based on risk stratification by SWG score

| **Cohort** | **Risk**  **stratification** | **No of**  **patients** | **Median**  **survival (months)** | **95% CI^*^**  **(months)** | **P value** |
| --- | --- | --- | --- | --- | --- |
| Present study | Low risk | 45 | 24**^.^**00 | 13**^.^**78-34**^.^**22 | <0**^.^**001 |
|  | High risk | 78 | 13**^.^**00 | 11**^.^**57-14**^.^**22 |  |
| TCGA | Low risk | 42 | 18**^.^**53 | 9**^.^**61-27**^.^**46 | 0**^.^**001 |
|  | High risk | 88 | 12**^.^**97 | 10**^.^**56-15**^.^**38 |  |

**^*^**CI- Confidence interval
