# Supplementary material for: A Fourteen Gene GBM Prognostic Signature Identifies Association of Immune Response Pathway and Mesenchymal Subtype with High Risk Group
Source: PLoS One. 2013 Apr 30;8(4):e62042. doi: 10.1371/journal.pone.0062042 (PMC3639942; doi:10.1371/journal.pone.0062042)
Supplement: Table S8 — The expression pattern of genes in low risk and high risk groups in the TCGA data set. (DOCX) [file pone.0062042.s010.docx]

**Supplementary table S8:** The expression pattern of genes in low risk and high risk groups in the TCGA data set

| **Sl No** | **Gene symbol** | **Low risk** | | | **High risk** | | |
| --- | --- | --- | --- | --- | --- | --- | --- |
|  |  | **Median** | **Mean** | **SD^*^** | **Median** | **Mean** | **SD^*^** |
| 1 | AGT | -0**^.^**013 | -0**^.^**112 | 1**^.^**483 | 0**^.^**296 | 0**^.^**096 | 1**^.^**303 |
| 2 | EGFR | 1**^.^**942 | 2**^.^**361 | 2**^.^**141 | 1**^.^**655 | 2**^.^**282 | 2**^.^**046 |
| 3 | CHI3L1 | 1**^.^**407 | 1**^.^**037 | 1**^.^**571 | 2**^.^**347 | 2**^.^**362 | 0**^.^**864 |
| 4 | SOD2 | 1**^.^**419 | 1**^.^**534 | 1**^.^**185 | 2**^.^**424 | 2**^.^**398 | 1**^.^**110 |
| 5 | CCL2 | -0**^.^**021 | -0**^.^**081 | 1**^.^**343 | 1**^.^**920 | 1**^.^**843 | 1**^.^**207 |
| 6 | IGFBPL1 | 0**^.^**187 | 0**^.^**323 | 0**^.^**707 | -0**^.^**139 | -0**^.^**028 | 0**^.^**417 |
| 7 | MBP | -2**^.^**546 | -2**^.^**666 | 1**^.^**072 | -1**^.^**779 | -1**^.^**992 | 1**^.^**082 |
| 8 | CPE | -1**^.^**629 | -1**^.^**733 | 1**^.^**205 | -1**^.^**614 | -1**^.^**509 | 0**^.^**893 |
| 9 | OLFM1 | -2**^.^**372 | -2**^.^**510 | 0**^.^**776 | -2**^.^**210 | -2**^.^**287 | 0**^.^**857 |
| 10 | MCF | -3**^.^**763 | -3**^.^**571 | 1**^.^**280 | -4**^.^**167 | -4**^.^**110 | 0**^.^**897 |
| 11 | PACSIN1 | -5**^.^**456 | -5**^.^**253 | 0**^.^**851 | -4**^.^**912 | -4**^.^**553 | 1**^.^**248 |
| 12 | CALCRL | 2**^.^**619 | 2**^.^**570 | 1**^.^**132 | 1**^.^**655 | 1**^.^**692 | 1**^.^**061 |
| 13 | SNCA | -5**^.^**202 | -4**^.^**967 | 0**^.^**963 | -4**^.^**070 | -4**^.^**071 | 0**^.^**765 |
| 14 | TOP2A | 3**^.^**614 | 3**^.^**512 | 1**^.^**134 | 2**^.^**828 | 2**^.^**835 | 0**^.^**945 |

*****SD - Standard deviation
