# Supplementary material for: A Fourteen Gene GBM Prognostic Signature Identifies Association of Immune Response Pathway and Mesenchymal Subtype with High Risk Group
Source: PLoS One. 2013 Apr 30;8(4):e62042. doi: 10.1371/journal.pone.0062042 (PMC3639942; doi:10.1371/journal.pone.0062042)
Supplement: Table S9 — List of differentially regulated genes between high risk and low risk patients derived from TCGA data set. (DOCX) [file pone.0062042.s011.docx]

| **Supplementary table S9:** List of differentially regulated genes between high risk and low risk patients derived from TCGA data set. | | | | | | |
| --- | --- | --- | --- | --- | --- | --- |
| **No.** | **Symbol** | **Gene name** | **High risk (mean)** | **Low risk (mean)** | **Adjusted**  **p value** | **Fold change** |
| 1 | CXCL14 | chemokine (C-X-C motif) ligand 14 | -0.73 | -2.94 | 0.00 | 2.21 |
| 2 | SAA1 | serum amyloid A1 | 5.14 | 2.95 | 0.03 | 2.19 |
| 3 | CCL2 | chemokine (C-C motif) ligand 2 | 1.84 | -0.08 | 0.00 | 1.92 |
| 4 | IL8 | interleukin 8 | 1.88 | -0.04 | 0.00 | 1.91 |
| 5 | SPP1 | secretedphosphoprotein 1 | 3.50 | 1.60 | 0.00 | 1.90 |
| 6 | LGALS3 | lectin, galactoside-binding, soluble, 3 | 2.16 | 0.36 | 0.05 | 1.80 |
| 7 | S100A8 | S100 calcium binding protein A8 | 1.93 | 0.21 | 0.00 | 1.73 |
| 8 | PTX3 | pentraxin-related gene, rapidly induced by IL-1 beta | 3.93 | 2.27 | 0.00 | 1.66 |
| 9 | S100A9 | S100 calcium binding protein A9 | 1.96 | 0.33 | 0.00 | 1.63 |
| 10 | BIRC3 | baculoviral IAP repeat-containing 3 | 0.53 | -1.05 | 0.00 | 1.59 |
| 11 | CYP1B1 | cytochrome P450, family 1, subfamily B, polypeptide 1 | -0.48 | -2.02 | 0.02 | 1.55 |
| 12 | CLDN23 | claudin 23 | 1.80 | 0.26 | 0.00 | 1.54 |
| 13 | CHI3L2 | chitinase 3-like 2 | 4.15 | 2.63 | 0.02 | 1.52 |
| 14 | CFD | complement factor D (adipsin) | 1.31 | -0.14 | 0.00 | 1.45 |
| 15 | CP | ceruloplasmin (ferroxidase) | 3.27 | 1.83 | 0.01 | 1.44 |
| 16 | SAA2 | serum amyloid A2 | 2.13 | 0.70 | 0.00 | 1.43 |
| 17 | PBEF1 | nicotinamidephosphoribosyltransferase | 3.14 | 1.75 | 0.02 | 1.39 |
| 18 | TIMP1 | TIMP metallopeptidase inhibitor 1 | 3.17 | 1.83 | 0.04 | 1.34 |
| 19 | CSTA | cystatin A (stefin A) | 3.46 | 2.12 | 0.00 | 1.34 |
| 20 | PLAU | plasminogen activator, urokinase | 3.31 | 1.99 | 0.00 | 1.32 |
| 21 | SPOCD1 | SPOC domain containing 1 | 4.05 | 2.75 | 0.04 | 1.30 |
| 22 | CD69 | CD69 molecule | 1.76 | 0.46 | 0.00 | 1.30 |
| 23 | AIF1 | allograft inflammatory factor 1 | 1.36 | 0.06 | 0.00 | 1.29 |
| 24 | ALDH1A3 | aldehyde dehydrogenase 1 family, member A3 | -0.33 | -1.61 | 0.00 | 1.29 |
| 25 | CD52 | CD52 molecule | 0.17 | -1.11 | 0.00 | 1.28 |
| 26 | TSLP | thymic stromal lymphopoietin | 1.72 | 0.44 | 0.00 | 1.28 |
| 27 | RAB34 | RAB34, member RAS oncogene family | 1.21 | -0.06 | 0.02 | 1.27 |
| 28 | NCF2 | neutrophil cytosolic factor 2 | 1.83 | 0.56 | 0.00 | 1.26 |
| 29 | ALOX5AP | arachidonate 5-lipoxygenase-activating protein | 1.27 | 0.01 | 0.00 | 1.26 |
| 30 | MS4A4A | membrane-spanning 4-domains, subfamily A, member 4 | 2.74 | 1.48 | 0.00 | 1.26 |
| 31 | APOC1 | apolipoprotein C-I | 2.97 | 1.72 | 0.00 | 1.25 |
| 32 | HAMP | hepcidin antimicrobial peptide | 1.41 | 0.15 | 0.00 | 1.25 |
| 33 | VDR | vitamin D (1,25- dihydroxyvitamin D3) receptor | 1.16 | -0.09 | 0.03 | 1.25 |
| 34 | BCL2A1 | BCL2-related protein A1 | 1.95 | 0.70 | 0.00 | 1.24 |
| 35 | BATF | basicleucine zipper transcription factor, ATF-like | 2.22 | 0.99 | 0.00 | 1.22 |
| 36 | HSD11B1 | hydroxysteroid (11-beta) dehydrogenase 1 | -2.02 | -3.23 | 0.00 | 1.22 |
| 37 | MARCO | macrophage receptor with collagenous structure | 0.03 | -1.18 | 0.00 | 1.21 |
| 38 | AQP9 | aquaporin 9 | -0.38 | -1.59 | 0.03 | 1.21 |
| 39 | RNASE2 | ribonuclease, RNase A family, 2 (liver, eosinophil-derived neurotoxin) | 2.53 | 1.32 | 0.00 | 1.21 |
| 40 | IFI30 | interferon, gamma-inducible protein 30 | 3.49 | 2.28 | 0.00 | 1.21 |
| 41 | IER3 | immediate early response 3 | 1.25 | 0.05 | 0.00 | 1.19 |
| 42 | CD53 | CD53 molecule | 1.65 | 0.47 | 0.00 | 1.18 |
| 43 | CXCL1 | chemokine (C-X-C motif) ligand 1 (melanoma growth stimulating activity, alpha) | -0.50 | -1.67 | 0.02 | 1.17 |
| 44 | CASP1 | caspase 1, apoptosis-related cysteine peptidase (interleukin 1, beta, convertase) | 2.62 | 1.45 | 0.00 | 1.17 |
| 45 | LY75 | CD302 molecule; lymphocyte antigen 75 | 1.40 | 0.24 | 0.01 | 1.17 |
| 46 | EMP1 | epithelial membrane protein 1 | 3.18 | 2.02 | 0.01 | 1.16 |
| 47 | FAS | Fas (TNF receptor superfamily, member 6) | 1.53 | 0.37 | 0.00 | 1.16 |
| 48 | MAL | mal, T-celldifferentiationprotein | -2.93 | -4.08 | 0.02 | 1.15 |
| 49 | FLJ22662 | Phospholipase B domain containing 1 | 1.54 | 0.39 | 0.01 | 1.15 |
| 50 | SLC31A2 | solute carrier family 31 (copper transporters), member 2 | -0.57 | -1.70 | 0.00 | 1.13 |
| 51 | DENND2D | DENN/MADD domain containing 2D | 2.13 | 1.01 | 0.00 | 1.12 |
| 52 | IL18 | interleukin 18 (interferon-gamma-inducingfactor) | 1.22 | 0.11 | 0.00 | 1.11 |
| 53 | FBP1 | fructose-1,6-bisphosphatase 1 | 1.35 | 0.24 | 0.00 | 1.11 |
| 54 | C1RL | complement component 1, r subcomponent-like | 1.79 | 0.68 | 0.02 | 1.10 |
| 55 | SERPINB1 | serpin peptidase inhibitor, clade B (ovalbumin), member 1 | 1.88 | 0.78 | 0.00 | 1.10 |
| 56 | APOC2 | apolipoprotein C-II | 0.88 | -0.22 | 0.00 | 1.09 |
| 57 | S100A10 | S100 calcium binding protein A10 | 2.46 | 1.38 | 0.02 | 1.08 |
| 58 | LYZ | lysozyme (renal amyloidosis) | 1.80 | 0.72 | 0.00 | 1.08 |
| 59 | CXCL3 | chemokine (C-X-C motif) ligand 3 | 1.20 | 0.12 | 0.01 | 1.08 |
| 60 | CD33 | CD33 molecule | 1.50 | 0.43 | 0.00 | 1.08 |
| 61 | UBD | ubiquitin D | 3.22 | 2.15 | 0.03 | 1.08 |
| 62 | SAMSN1 | SAM domain, SH3 domain and nuclear localization signals 1 | 1.49 | 0.42 | 0.01 | 1.07 |
| 63 | SQRDL | sulfidequinonereductase-like (yeast) | 1.80 | 0.73 | 0.00 | 1.07 |
| 64 | OSM | oncostatin M | 0.84 | -0.21 | 0.00 | 1.06 |
| 65 | CTSZ | cathepsin Z | 0.87 | -0.17 | 0.04 | 1.05 |
| 66 | S100A11 | S100 calcium binding protein A11; S100 calcium binding protein A11 pseudogene | 2.20 | 1.15 | 0.00 | 1.05 |
| 67 | CCL20 | chemokine (C-C motif) ligand 20 | 1.76 | 0.71 | 0.01 | 1.05 |
| 68 | ZFP36 | zinc finger protein 36, C3H type, homolog (mouse) | 1.21 | 0.18 | 0.01 | 1.03 |
| 69 | HAVCR2 | hepatitis A viruscellular receptor 2 | 1.55 | 0.52 | 0.00 | 1.03 |
| 70 | C3 | similar to Complement C3 precursor; complement component 3 | 1.95 | 0.93 | 0.04 | 1.02 |
| 71 | SERPINE1 | serpin peptidase inhibitor, clade E (nexin, plasminogen activator inhibitor type 1), member 1 | 2.14 | 1.12 | 0.01 | 1.02 |
| 72 | CXCR4 | chemokine (C-X-C motif) receptor 4 | 2.87 | 1.86 | 0.01 | 1.01 |
| 73 | SRGN | serglycin | 1.24 | 0.24 | 0.00 | 1.00 |
| 74 | GNG4 | guanine nucleotide binding protein (G protein), gamma 4 | -1.03 | 0.02 | 0.03 | -1.05 |
| 75 | MEX3A | mex-3 homolog A (C. elegans) | 2.49 | 3.58 | 0.03 | -1.09 |
| 76 | DCX | doublecortin | -1.12 | 0.59 | 0.04 | -1.71 |
