# Supplementary material for: A Fourteen Gene GBM Prognostic Signature Identifies Association of Immune Response Pathway and Mesenchymal Subtype with High Risk Group
Source: PLoS One. 2013 Apr 30;8(4):e62042. doi: 10.1371/journal.pone.0062042 (PMC3639942; doi:10.1371/journal.pone.0062042)
Supplement: Table S10 — Transcript levels of 14 genes in four glioma subtypes. (DOCX) [file pone.0062042.s012.docx]

**Supplementary table S10** – Transcript levels of 14 genes in four glioma subtypes

| **Genes** | **Proneural** | **Neural** | **Classical** | **Mesenchymal** | **Expression Difference*** | **p value#** |
| --- | --- | --- | --- | --- | --- | --- |
| **TOP2A** | 3.81 ± 1.13 | 2.80 ± 102 | 2.94 ± 0.59 | 2.51 ± 0.72 | -1.30 | 0.000 |
| **CALCRL** | 2.78 ± 1.03 | 1.65 ± 1.07 | 1.19 ± 1.13 | 1.96 ± 0.99 | -0.82 | 0.004 |
| **MCF2** | -3.40 ± 1.16 | -3.56 ± 0.86 | -4.68 ± 0.73 | -4.11 ± 0.90 | -0.71 | 0.005 |
| **IGFBPL1** | 0.52 ± 0.65 | -0.30 ± .27 | -0.15 ± 0.25 | 0.00 ± 0.42 | -0.52 | 0.002 |
| **CCL2** | 0.03 ± 1.47 | 1.49 ± 1.19 | 0.78 ± 1.17 | 2.36 ± 1.12 | 2.33 | 0.000 |
| **CHI3L1** | 0.72 ± 1.46 | 1.92 ± 1.09 | 2.28 ± 0.50 | 2.73 ± 0.61 | 2.01 | 0.000 |
| **SOD2** | 1.23 ± 1.00 | 2.07 ± 0.73 | 1.85 ± 1.05 | 2.95 ± 1.07 | 1.60 | 0.000 |
| **EGFR** | 1.04 ± 1.65 | 2.62 ± 1.58 | 4.47 ± 1.68 | 1.62 ± 1.55 | 0.58 | 0.131 |
| **CPE** | -1.84 ± 0.93 | -1.06 ± 0.69 | -1.63 ± 1.01 | -1.56 ± 1.13 | 0.28 | 0.257 |
| **SNCA** | -4.43 ± 1.12 | -3.66 ± 0.94 | -4.82 ± 0.77 | -4.25 ± 0.70 | 0.18 | 0.411 |
| **MBP** | -2.00 ± 1.04 | -1.72 ± 0.92 | -3.27 ± 0.78 | -1.85 ± 1.06 | 0.15 | 0.559 |
| **AGT** | -0.39 ± 1.42 | 0.29 ± 0.99 | 0.94 ± 0.74 | -0.65 ± 1.42 | -0.26 | 0.450 |
| **PACSIN1** | -4.74 ± 1.24 | -3.76 ± 1.03 | -5.02 ± 0.94 | -5.26 ± 1.05 | -0.52 | 0.039 |
| **OLFM1** | -2.06 ± 0.88 | -1.82 ± 0.42 | -2.54 ± 0.62 | -2.92 ± 0.85 | -0.86 | 0.000 |

* Difference between Mesenchymal and Proneural subtypes as these two groups are enriched in High and Low risk respectively.

# Statistical significance between transcript levels of 14 genes between Proneural and Mesenchymal calculated by t test shown.
